# An RNA-dependent and phase-separated active subnuclear compartment safeguards repressive chromatin domains

**DOI:** 10.1016/j.molcel.2024.03.015

**Published:** 2024-05-02

**Authors:** Luigi Lerra, Martina Panatta, Dominik Bär, Isabella Zanini, Jennifer Yihong Tan, Agnese Pisano, Chiara Mungo, Célia Baroux, Vikram Govind Panse, Ana C. Marques, Raffaella Santoro

**Affiliations:** 1 Department of Molecular Mechanisms of Disease (DMMD), University of Zurich, Zurich 8057, Switzerland; 2 RNA Biology Program, Life Science Zurich Graduate School, University of Zurich, Zurich 8057, Switzerland; 3 Department of Computational Biology, University of Lausanne, Lausanne 1015, Switzerland; 4 Institute of Medical Microbiology, University of Zurich, Zurich 8057, Switzerland; 5 Molecular Life Science Program, Life Science Zurich Graduate School, University of Zurich, Zurich 8057, Switzerland; 6 Department of Plant and Microbial Biology and Zurich-Basel Plant Science Center, University of Zurich, Zurich 8057, Switzerland

**Keywords:** chromatin, nuclear condensates, phase separation, BAZ2A, H3K27me3, RNA, Malat1, nuclear speckles, ground-state pluripotency

## Abstract

The nucleus is composed of functionally distinct membraneless compartments that undergo phase separation (PS). However, whether different subnuclear compartments are connected remains elusive. We identified a type of nuclear body with PS features composed of BAZ2A that associates with active chromatin. BAZ2A bodies depend on RNA transcription and BAZ2A non-disordered RNA-binding TAM domain. Although BAZ2A and H3K27me3 occupancies anticorrelate in the linear genome, in the nuclear space, BAZ2A bodies contact H3K27me3 bodies. BAZ2A-body disruption promotes BAZ2A invasion into H3K27me3 domains, causing H3K27me3-body loss and gene upregulation. Weak BAZ2A-RNA interactions, such as with nascent transcripts, promote BAZ2A bodies, whereas the strong binder long non-coding RNA (lncRNA) *Malat1* impairs them while mediating BAZ2A association to chromatin at nuclear speckles. In addition to unraveling a direct connection between nuclear active and repressive compartments through PS mechanisms, the results also showed that the strength of RNA-protein interactions regulates this process, contributing to nuclear organization and the regulation of chromatin and gene expression.

## Highlights

• BAZ2A forms condensates around active chromatin of mouse embryonic stem cells
• BAZ2A bodies sequester BAZ2A and limit its invasion into H3K27m3 chromatin domains
• BAZ2A condensates depend on transcription and weak and transient interaction with RNA
• The lncRNA *Malat1* strongly associates with BAZ2A and impairs BAZ2A-body formation

## Graphical abstract

**Figure:**
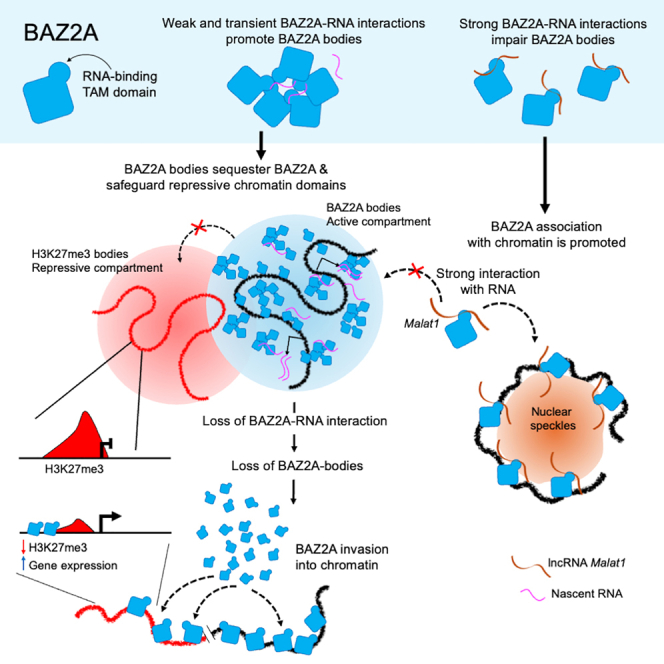

## Teaser

Lerra, Panatta, et al. showed that BAZ2A forms an active subnuclear compartment that protects repressive H3K27me3 chromatin using phase-separation mechanisms. BAZ2A bodies are mediated by RNAs with weak interaction to BAZ2A RNA-binding domain TAM, whereas strong RNA binders, such as *Malat1*, impair condensates while promoting BAZ2A association to chromatin.

## Introduction

Intracellular compartmentalization is a key feature of living organisms that allows spatial and temporal regulation of biological processes. In the eukaryotic nucleus, the DNA is hierarchically organized into nucleosomes, chromatin fibers, loops, topologically associating domains, and compartments.^1^ Moreover, the nucleus is composed of several membraneless compartments, also referred to as nuclear bodies or condensates, which serve specialized functions such as the nucleolus for ribosome biogenesis.^2^ These condensates compartmentalize and concentrate proteins and RNA molecules required for each process, typically at specific genomic loci, to allow a much greater efficiency of reactions.^3^^,^^4^^,^^5^ It has been proposed that these nuclear subcompartments originate via a mechanism of phase separation (PS) that is mainly driven by the multivalency of proteins and RNAs, which create a network of homo- and heterotypic interactions.^2^^,^^5^^,^^6^^,^^7^^,^^8^ The nature and strength of these interactions also regulate the physiochemical properties of the compartments. Low-affinity and transient interactions induce liquid-like properties. By contrast, strong and stable interactions reduce the mobility of proteins and RNAs, thereby causing the formation of gel– and solid-like PS compartments.^9^ The building blocks for PS are usually proteins with intrinsically disordered regions (IDRs), such as FUS,^10^ TDP-43,^11^ Med1,^12^ or HP1.^13^^,^^14^ IDRs are considered responsible for transient and multivalent interactions with other biomolecules and can be key drivers of PS.^15^ IDRs can also associate with RNA, which is a powerful regulator since its high negative charge can affect the formation and properties of PS compartments.^16^^,^^17^ The effects of diverse RNAs on transcriptional condensates also suggest that they should act in a sequence-independent manner.^18^ Thus, nuclear bodies are tightly regulated structures, and the absence of a surrounding membrane allows their components to be highly dynamic, thereby regulating biological processes in space and time. However, while numerous studies are beginning to reveal the mechanisms of formation and functions of singular nuclear compartments, whether and how different nuclear condensates can be functionally interconnected remains unexplored. Moreover, it remains unclear whether specific RNA sequences can have an impact on nuclear condensates in cells.

In this work, we have provided such an example by showing that an active subnuclear compartment in mouse embryonic stem cells (ESCs) protects repressive chromatin compartments using PS mechanisms that are promoted or impaired according to the strength of RNA interactions with a non-disordered RNA-binding protein. We identified in ESCs a type of nuclear body composed of BAZ2A (also known as TIP5). BAZ2A is an RNA-binding protein that in differentiated cells localizes in nucleoli and associates with the long non-coding RNA (lncRNA) pRNA and ribosomal RNA (rRNA) genes.^19^^,^^20^ However, in ESCs, the nucleolar function of BAZ2A is abrogated due to the lack of pRNA maturation.^21^^,^^22^ Instead, BAZ2A associates with large active chromatin domains and regulates gene expression and H3K27me3 only in ground-state pluripotent ESCs (i.e., ESC cultured with “2i” MEK/ERK and GSK3 inhibitors ESC + 2i^23^), but not in developmentally advanced ESCs (ESC + serum),^24^ highlighting distinct chromatin features according to developmental stage. However, it remained elusive how BAZ2A associates with active chromatin domains while regulating H3K27me3 repressive domains, which are not bound by BAZ2A.

## Results

### BAZ2A forms bodies that depend on RNA and its RNA-binding domain TAM

**Figure 1:**
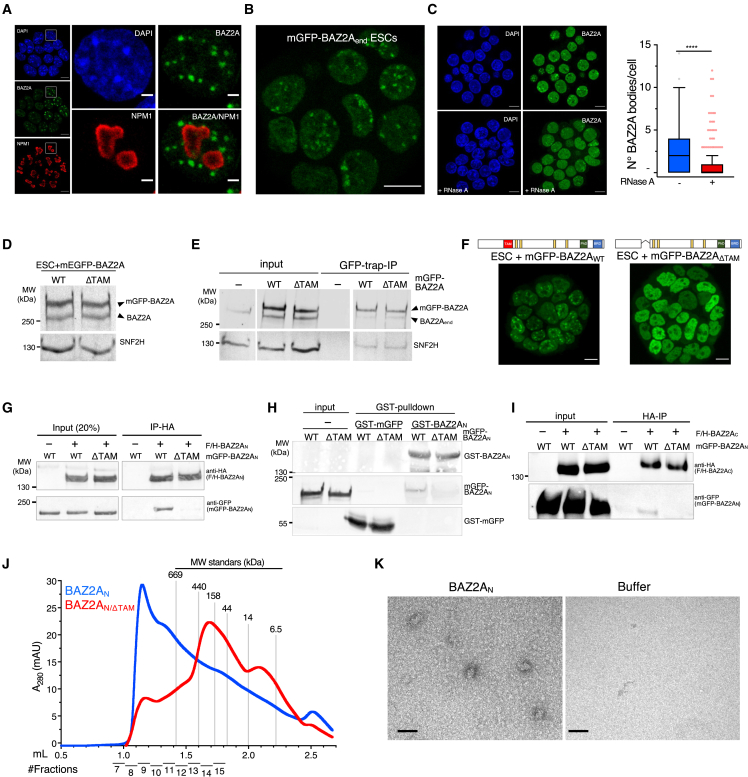
BAZ2A forms bodies that depend on its RNA-binding domain TAM (A) Representative immunofluorescence image showing BAZ2A localization in ESC + 2i using antibodies against BAZ2A and the nucleolar marker NPM1. Scale bars, left panel: 10 μm; right panel: 2 μm. (B) Live-cell image of ESCs expressing endogenous BAZ2A tagged with mGFP (mGFP-BAZ2A_end_). Scale bars: 10 μm. (C) Images of ESCs treated with RNase A. Boxplot shows the number of BAZ2A bodies/cell. Error bars represent SD. Statistical significance for three independent experiments was calculated using Mann-Whitney test (^∗∗∗^p < 0.001). (D) Western blot showing the expression levels of endogenous BAZ2A and mGFP-BAZ2A_WT_ and -BAZ2A_ΔTAM_ transgenes in the corresponding ESC lines. BAZ2A signal was detected with BAZ2A antibodies. SNF2H serves as loading control. (E) GFP-trap immunoprecipitation (IP) in parental ESCs, ESC + mGFP-BAZ2A_WT_, and ESC + mGFP- BAZ2A_ΔTAM_. BAZ2A signal was detected with BAZ2A antibodies. SNF2H is a known BAZ2A-interacting protein. (F) Live-cell image of ESC + mGFP-BAZ2A_WT_ and ESC + mGFP- BAZ2A_ΔTAM_. The domain composition of BAZ2A_WT_ (1,889 aa) and BAZ2A_ΔTAM_ is shown. Yellow lanes represent AT-hook domains. (G) Anti-HA immunoprecipitation from HEK293T cells transfected with plasmids expressing F/H-BAZ2A_N_ and mGFP-BAZ2A_N/WT_ or -BAZ2A_N/ΔTAM_. (H) GST pull-down of recombinant GST-BAZ2A_N/WT_ and mGFP-BAZ2A_N/WT_ or -BAZ2A_N/ΔTAM_. GST and GFP antibodies were used to visualize the corresponding proteins. (I) Anti-HA immunoprecipitation from HEK293T cells transfected with plasmids expressing F/H-BAZ2A_C_ and mGFP-BAZ2A_N/WT_ or -BAZ2A_N/ΔTAM_. (J) Profile of size exclusion chromatography of recombinant BAZ2A_N_ and BAZ2A_N/ΔTAM_. Fractions were measured by absorbance at 280 nm. (K) Negative staining of BAZ2A_N_ fractionated sample corresponding to >600 kDa. Scale bars: 50 nm.

To understand how BAZ2A bound to active chromatin regulates repressive chromatin domains in ESC + 2i, we performed immunofluorescence (IF) analysis and found that BAZ2A displays a pattern of nucleoplasmic puncta that resemble bodies (Figure 1A). We observed a similar pattern by live-cell imaging using an ESC line expressing endogenous BAZ2A tagged with monomeric GFP (mGFP) (ESC + mGFP-BAZ2A_end_) (Figures 1B and S1A). ESC + serum also showed a similar pattern (Figure S1B). Consistent with previous studies, BAZ2A did not colocalize within nucleoli of both ESC types.^22^^,^^24^ We noticed that only about 50% of ESCs contained large BAZ2A bodies (Figure S1C). This heterogeneity was not due to distinct cell cycle phases, except for mitotic cells, which were depleted of BAZ2A bodies (Figures S1D and S1E). However, we cannot exclude that those cells lacking large BAZ2A bodies might contain small BAZ2A bodies that are undetectable under standard confocal microscopy. Segmentation of IF images identified nucleoplasmic 3D-spot objects of 300 nm diameter, which were not included in BAZ2A bodies (Figure S1G). BAZ2A levels in bodies were 100-fold higher than in spots. We also observed some fusion events, suggesting that BAZ2A bodies display some degree of mobility (Figure S1F; Video S1). Fluorescence recovery after photobleaching (FRAP) of mGFP-BAZ2A bodies revealed that 35% of BAZ2A showed moderate mobility (22 s half-time of photobleaching recovery, Figure S1H), which is consistent with previous work showing that a large fraction of BAZ2A associates with chromatin in ESCs.^24^ Accordingly, Triton X-100 pre-extraction of proteins not bound to chromatin prior imaging showed about 30% loss of BAZ2A signal in bodies compared with untreated cells (Figure S1I). These results suggest that BAZ2A bodies correspond to BAZ2A-bound to chromatin surrounded by BAZ2A moieties not associated with chromatin. Finally, BAZ2A bodies depend on RNA since treatment with Rnase A significantly decreased their number (Figure 1C).

Video S1. The video illustrates fusion events of BAZ2A bodies in ESC + 2i, related to Figure 1

We asked whether BAZ2A-body formation might occur through mechanisms involving BAZ2A self-assembly. We performed GFP-trap immunoprecipitation (IP) in an ESC line ectopically expressing mGFP-tagged BAZ2A at levels similar to endogenous BAZ2A (ESC + mGFP-BAZ2A_WT_; Figures 1D and 1E). mGFP-BAZ2A could interact with the untagged endogenous BAZ2A, indicating that BAZ2A can self-assemble in ESCs. BAZ2A self-interaction could also be observed in HEK293T cells (Figure S1J). BAZ2A contains an RNA-binding domain named TAM (TIP5/ARBD/MBD).^19^^,^^20^ Given the implications of RNA in BAZ2A bodies, we analyzed the role of BAZ2A-TAM domain in BAZ2A-body formation in an ESC line ectopically expressing a BAZ2A mutant lacking the TAM domain (deletion aa 506–559 mGFP-BAZ2A_ΔTAM_) that expresses at comparable levels to endogenous BAZ2A and ectopically expressed mGFP-BAZ2A_WT_ (Figure 1D). Live-cell imaging showed that mGFP-BAZ2A_ΔTAM_ could not form BAZ2A bodies, indicating that the TAM domain is required for their formation (Figure 1F). Impairment of BAZ2A bodies could also be observed in ESCs expressing mGFP-BAZ2A_W551G/Y552A_ that was previously shown to not associate with RNA^19^ (Figure S1K). GFP-trap IP, however, showed that mGFP-BAZ2A_ΔTAM_ could still associate with endogenous BAZ2A (Figure 1E). These results suggest that other BAZ2A regions than TAM domain could mediate BAZ2A self-interaction in cells, however, these interactions are not sufficient for the formation of BAZ2A bodies. To identify which BAZ2A domain mediates self-interaction, we performed Ips in HEK293T cells transfected with plasmids expressing BAZ2A N- and C-terminal regions tagged with FLAG and hemagglutinin (HA) (F/H) or mGFP (BAZ2A_N_ aa 1–744; BAZ2A_C_ aa 744–1,854) (Figures 1G–1I). F/H-BAZ2A_N_ and mGFP-BAZ2A_N_ could interact with each other, and this interaction was drastically reduced with BAZ2A_N/ΔTAM_ mutant (Figure 1G). Glutathione S-transferase (GST) pull-down using recombinant proteins showed that BAZ2A N-terminal self-interaction is direct and dependent on the TAM domain (Figure 1H). In cells, BAZ2A self-interaction could also occur between the C terminus (Figure S1L). Although to a less extent, BAZ2A_N_ and BAZ2A_C_ could interact with each other, and this association also depended on TAM domain (Figure 1I). Thus, BAZ2A-self-assembly in the absence of TAM domain is most likely occurring through its C-terminal domain; however, this interaction is not sufficient to form BAZ2A bodies, which might probably involve multimeric BAZ2A interactions. To support these data, we performed size exclusion chromatography (SEC) using recombinant BAZ2A_N_ and BAZ2A_N/ΔTAM_. SEC profiles showed that BAZ2A_N_ forms supramolecular complexes larger than 600 kDa, which should include four up to six units of BAZ2A_N_ (Figure 1J). Gel electrophoresis analysis of SEC fractionated samples showed that these large BAZ2A_N_ complexes could enter into the gel only upon denaturation conditions (Figure S1M). The formation of higher-order complexes was supported by negative staining of fractionated BAZ2A_N_ samples that displayed an oval shape with diameter of 40 nm size (Figure 1K). By contrast, SEC detected only BAZ2A_N/ΔTAM_ monomers that could run into gel electrophoresis under native conditions (Figures 1J and S1K). Thus, TAM domain is required for the formation of BAZ2A supramolecular complexes.

**Figure 2:**
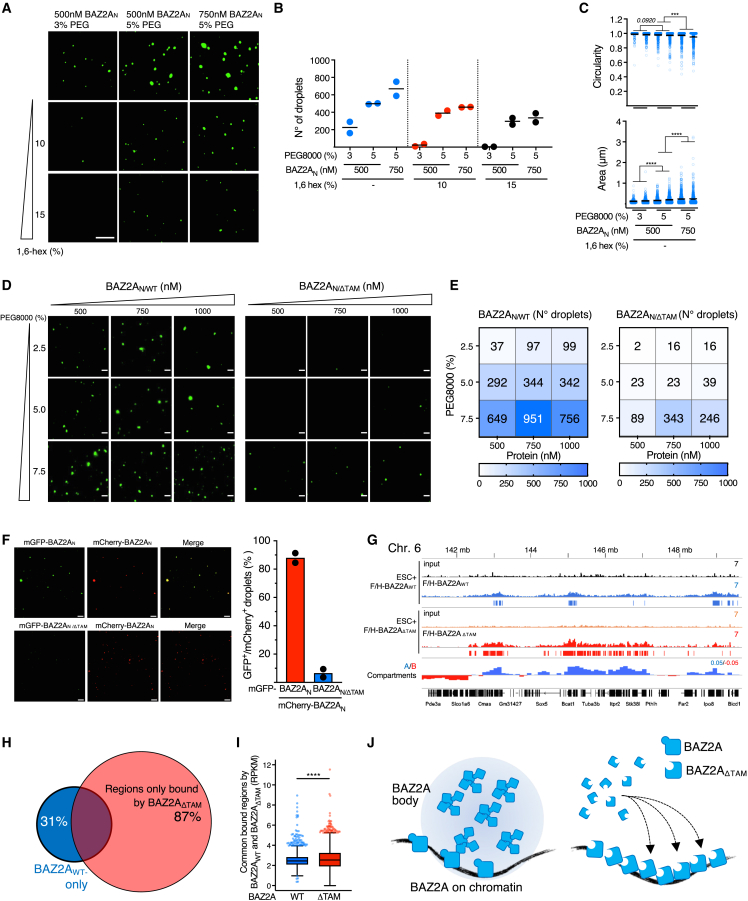
BAZ2A bodies in ESCs show physicochemical LLPS properties *in vitro* (A) Representative images of recombinant mGFP-BAZ2A_N_ under the indicated conditions. Scale bars: 5 μm. (B and C) Quantification of the number (B) and circularity and area (C) of recombinant BAZ2A_N_ droplets from three independent experiments. (D and E) Representative images (D) and corresponding quantifications of droplets number (E) of mGFP-BAZ2A_N/WT_ and mGFP-BAZ2A_N/ΔTAM_. Scale bars: 5 μm. (F) Left panel, representative images of droplets formed with recombinant mCherry-BAZ2A_N/WT_ and mGFP-BAZ2A_N/WT_ or mGFP-BAZ2A_N/ΔTAM_. Scale bars: 2 μm. Right panel, quantifications of mGFP- and mCherry-positive droplets from two independent experiments. (G) Tracks displaying BAZ2A_WT_ and BAZ2A_ΔTAM_ occupancy in ESC + F/H-BAZ2Ar_WT_ and ESC + F/H-BAZ2Ar_ΔTAM_. Eigenvector values of A and B compartments in ESC + 2i are from Dalcher et al.^24^ (H) Proportional Venn diagram showing common and specific genome occupancy of BAZ2A_WT_ and BAZ2A_ΔTAM_. (I) Boxplots showing levels of BAZ2A_WT_ and BAZ2A_ΔTAM_ occupancy at domains both bound by BAZ2A_WT_ and BAZ2A_ΔTAM_. Error bars represent SD. Statistical significance was calculated using Mann-Whitney test (^∗∗∗∗^p < 0.0001). (J) Model showing BAZ2A_WT_ forming bodies through the TAM domain and the association with chromatin. The lack of the TAM domain impairs body formation and promotes BAZ2A association with chromatin.

To determine whether BAZ2A has physicochemical properties that may contribute to bodies formation, we examined recombinant mGFP-BAZ2A_N_ using *in vitro* droplet assays (Figures 2 and S2A). mGFP-BAZ2A_N_ formed spherical droplets that displayed properties consistent with PS liquid condensates, including sensitivity to the concentration of proteins, 16-hexanediol polyethylene glycol, and salt, all affecting number of droplets circularity and area (Figures 2A–2E and S2B–S2D). By contrast, BAZ2A_N/ΔTAM_ could not form droplets under the conditions used for BAZ2A_N/WT_ (Figures 2D and 2E). Moreover, by incubating together recombinant mCherry-BAZ2A_N_ and mGFP-BAZ2A_N/ΔTAM_, droplets were only formed by mCherry-BAZ2A_N_ (Figure 2F).

To determine which factors can associate with BAZ2A bodies, we applied a recent protocol for the identification of proteins able to partition into condensates.^25^ Recombinant BAZ2A droplets formed into a soluble nuclear extract were isolated by centrifugation, and the pellet, which contains proteins partitioned into BAZ2A condensates, was analyzed by mass spectrometry (Figure S3A). As expected, the amount of recombinant BAZ2A_WT_ in the pellet was much higher than BAZ2A_ΔTAM_, indicating the presence of BAZ2A bodies in the pellet (Figure S3B). We identified 54 proteins that were significantly partitioned into BAZ2A condensates (Figure S3C; Table S1). Only 11 of them were also significantly enriched in BAZ2A_ΔTAM_ pellet, including BAZ2A_ΔTAM_ that was, however, much less enriched compared with BAZ2A_WT_ samples (BAZ2A_WT_/Control: 39.4; BAZ2A_ΔTAM_/Control: 2.8; Figure S3D). Gene ontology analysis of proteins specifically present within BAZ2A condensates showed a significant enrichment in processes linked to gene expression and regulation of chromosome organization (Figure S3E). Among these proteins, we found positive regulators of RNA polymerase II (RNA Pol II) transcription, such as BRD3, BRD4, SUPT6H, and RAD21, indicating that BAZ2A bodies not only sequester BAZ2A but also can attract other chromatin regulators. Finally, a large majority of these proteins displayed high IDR content (Figure S3F), suggesting that their partitioning into BAZ2A bodies can be mediated by PS features.

To determine whether BAZ2A bodies depend on BAZ2A association with chromatin, we performed quantitative FLAG-chromatin immunoprecipitation sequencing (ChIP-seq) in ESC lines ectopically expressing F/H-BAZ2A_WT_ or F/H-BAZ2A_ΔTAM_ (Figures 2G–2I). Both ESC lines expressed similar levels of BAZ2A, which was modified to be resistant to small interfering RNA (siRNA)-BAZ2A (ESC + F/H-BAZ2Ar_WT_ or F/H-BAZ2Ar_ΔTAM_) in order to specifically downregulate endogenous BAZ2A expression (Figure S2E). Consistent with previous results, BAZ2A_WT_ associates with large active chromatin domains that correspond to active A compartments^24^ (Figure 2G). BAZ2A_ΔTAM_ also associated with chromatin; however, its occupancy was significantly higher compared with BAZ2A_WT_ (Figures 2G–2I), suggesting that the lack of TAM domain and the consequent impairment of BAZ2A bodies promote aberrant BAZ2A association with chromatin (Figure 2J). Together, the results indicate that BAZ2A-TAM domain mediates the formation of BAZ2A bodies, which have physicochemical LLPS properties *in vitro*, and the formation of these bodies impacts BAZ2A association with chromatin.

### BAZ2A bodies associate with and regulate H3K27me3 bodies

**Figure 3:**
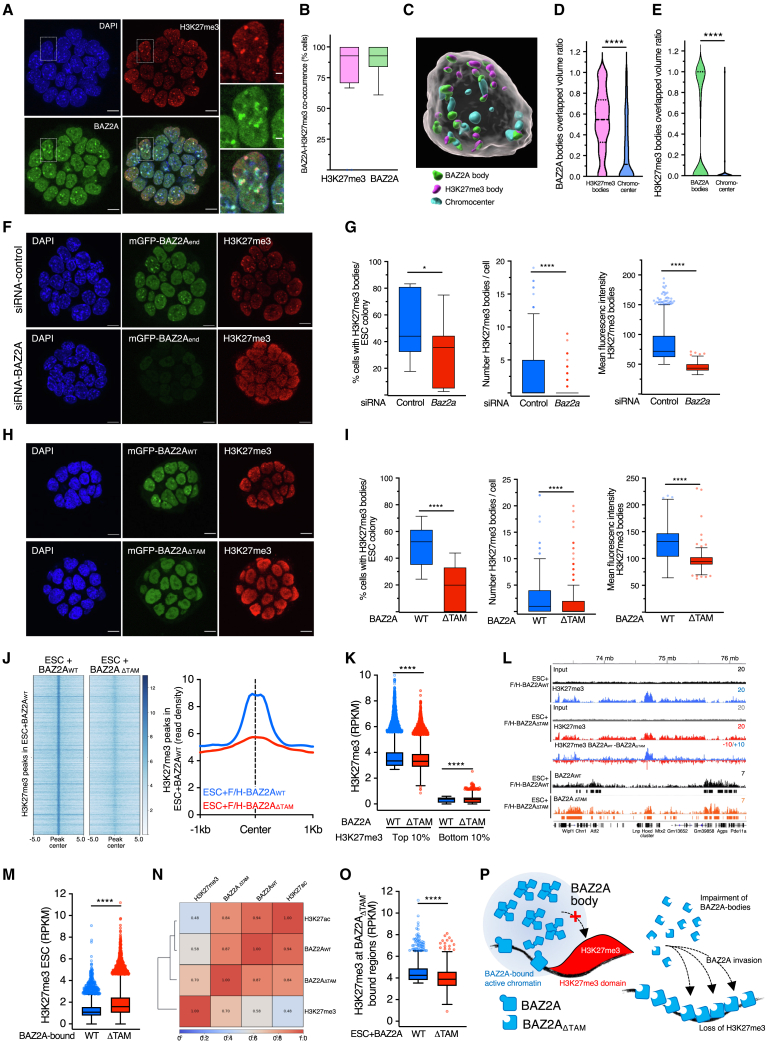
BAZ2A bodies associate and regulate H3K27me3 bodies (A) Representative immunofluorescence images showing BAZ2A and H3K27me3 distribution in ESC + 2i. Right images represent the magnification of the section labeled with a rectangle. Scale bars, left images: 10 μm; right images: 2 μm. (B) Proportion of ESCs with H3K27me3 bodies having BAZ2A bodies (left) and with BAZ2A bodies having H3K27me3 bodies (right panel). Data were from 20 ESC colonies. (C) 3D image of one ESC showing BAZ2A and H3K27me3 bodies and DAPI-stained chromocenters. (D and E) Quantification of overlapping volume of BAZ2A bodies over H3K27me3 bodies and chromocenters (D) and of H3K27me3 bodies over BAZ2A bodies and chromocenters (E). Data are from 13 nuclei. Statistical significance was calculated with Mann-Whitney test (^∗∗∗∗^p < 0.0001). (F) Representative immunofluorescence images showing mGFP-BAZ2A_end_ and H3K27me3 distribution in ESC depleted of BAZ2A by siRNA. Scale bars: 10 μm. (G) Quantification of ESCs with H3K27me3 bodies and number and mean intensity of H3K27me3 bodies upon treatment with siRNA-Control and siRNA-*Baz2a*. Error bars represent SD. (H) Representative immunofluorescence images showing BAZ2A and H3K27me3 distribution in ESC + mGFP-BAZ2A_WT_ and ESC + mGFP-BAZ2A_ΔTAM_. Scale bars: 10 μm. (I) Quantification of ESCs with H3K27me3 bodies, and the number and mean intensity of H3K27me3 bodies in ESCs expressing mGFP-BAZ2A_WT_ and mGFP-BAZ2A_ΔTAM_. Data are from three independent experiments. (J) Left panel, heatmap showing H3K27me3 peaks detected in ESC + F/H-BAZ2Ar_WT_ and the corresponding signal in ESC + F/H-BAZ2Ar_ΔTAM_. Right panel, average density plots of H3K27me3-ChIP-seq read counts at ±1 kb from H3K27me3 peak summits in the corresponding ESC lines. (K) Levels of H3K27me3 at the 10% top or bottom H3K27me3 regions in ESC + BAZ2A_WT_ and the corresponding levels in ESC + BAZ2A_ΔTAM_. Values are shown as average reads per kilobase per million (RPKM) of a 10-kb bin size region. (L) Tracks displaying H3K27me3 BAZ2A_WT_ and BAZ2A_ΔTAM_ occupancy in ESC + F/H-BAZ2Ar_WT_ and ESC + F/H-BAZ2Ar_ΔTAM_ at *Hoxd* gene cluster. (M) H3K27me3 levels at BAZ2A_WT_- and BAZ2A_ΔTAM_-bound regions in parental ESC + 2i. (N) Spearman’s correlation heatmap for BAZ2A_WT_, BAZ2A_ΔTAM_, H3K27ac, and H3K27me3. H3K27me3 and H3K27ac ChIP-seq in ESC + 2i were from Dalcher et al.^24^ (O) H3K27me3 levels in ESC + F/H-BAZ2Ar_WT_ and ESC + F/H-BAZ2Ar_ΔTAM_ at the 10% top H3K27me3 regions bound by BAZ2A_ΔTAM_. (P) Model showing BAZ2A_WT_ forming bodies through the TAM domain and the association with chromatin depleted of H3K27me3. The lack of the TAM domain impairs body formation and promotes BAZ2A invasion into H3K27me3 domains and the loss of this repressive signature. Statistical significance in boxplots (G), (I), (K), (M), and (O) was calculated with Mann-Whitney test (^∗^p < 0.05, ^∗∗∗∗^p < 0.0001). Error bars represent SD.

To determine where BAZ2A bodies localize in cells, we performed Ifs for H3K27me3 since previous work showed that BAZ2A regulates H3K27me3 genome occupancy specifically in ESC + 2i.^24^ In addition to a regular widespread nuclear distribution, H3K27me3 signal was also enriched in a discrete number of densely stained regions that colocalize with Polycomb repressive complex 2 (PRC2) component EZH2 and partially with PRC1 component RING1B (Figures 3A and S4A). H3K27me3 bodies were specific to ESC + 2i and not found in ESC + serum, consistent with a previous study (Figure S4B).^26^ Similarly to BAZ2A, H3K27me3 bodies were not detected in all cells (Figure 3A). Yet, 80% of cells showing BAZ2A bodies contained H3K27me3 bodies, and reciprocally, 90% of cells showing H3K27me3 bodies also formed BAZ2A bodies (Figure 3B). This indicates a strong co-occurrence of these nuclear compartments. Similarly to BAZ2A, treatment with Rnase A significantly affected H3K27me3 bodies, indicating their RNA dependency (Figure S4C).

3D images segmented for BAZ2A H3K27me3 and the DAPI-stained chromocenters^27^ revealed a frequent overlap of BAZ2A and H3K27me3 bodies (59%), whereas BAZ2A bodies overlap with chromocenters was less frequent (18%) (Figures 3C–3E and S4D). For H3K27me3 bodies, we found one fraction overlapping with BAZ2A bodies and the other with no or little overlap, whereas there was no overlap between H3K27me3 bodies and chromocenters (Figure 3E). These results indicate a frequent spatial proximity between BAZ2A and H3K27me3 bodies, a surprising result since previous ChIP-seq data in ESC + 2i showed that BAZ2A genomic occupancy anticorrelates with H3K27me3-marked chromatin^24^ (Figure 3N). Thus, active chromatin domains marked by BAZ2A and H3K27me3 repressive domains frequently occupy the same nuclear space, but their linear genome occupancy is different.

To determine whether H3K27me3 bodies depend on BAZ2A bodies, we performed Ifs in ESC + 2i depleted of BAZ2A by siRNA or expressing mGFP-BAZ2A_ΔTAM_ (Figures 3F–3I). In both cases, we found a significant reduction of cells with H3K27me3 bodies and number of H3K27me3 bodies/cell, indicating that H3K27me3 bodies depend on BAZ2A-TAM domain, which in turn is required for BAZ2A bodies. To test whether BAZ2A bodies regulate H3K27me3, we performed quantitative H3K27me3-ChIP-seq in ESC + F/H-BAZ2Ar_WT_ and F/H-BAZ2Ar_ΔTAM_ (Figures 3J–3O). BAZ2A_ΔTAM_ did not affect global mRNA and protein levels of EZH2 and RING1B and had only a minor effect on H3K27me3 (ca. 10% reduction) (Figures S4E and S4F; Table S2). Remarkably, BAZ2A_ΔTAM_ induced a global redistribution of H3K27me3. Specifically, H3K27me3 enriched domains identified in control cells decreased in signal upon BAZ2A_ΔTAM_ expression, whereas regions with low H3K27me3 levels in control cells increased this repressive mark (Figures 3J, 3K, and S4G). However, this H3K27me3 gain was low and, in general, did not result in the formation of new H3K27me3 peaks. This BAZ2A_ΔTAM_-mediated redistribution of H3K27me3 was similar to the reported alterations detected in BAZ2A-depleted ESCs (Figure S4H),^24^ indicating that BAZ2A-mediated regulation of H3K27me3 requires the TAM domain. BAZ2A_ΔTAM_-bound regions corresponded to regions that in parental ESC + 2i are enriched in H3K27me3 compared with BAZ2A_WT_-bound regions (Figures 3L–3N). For example, BAZ2A_ΔTAM_ associates with H3K27me3-marked *Hoxd* gene cluster that is not bound by BAZ2A_WT_ (Figure 3l). Moreover, BAZ2A_ΔTAM_-bound regions showed reduced H3K27me3 levels in ESC + BAZ2A_ΔTAM_ relative to ESC + BAZ2A_WT_ (Figure 3O). These results suggest that when BAZ2A is not sequestered in bodies, it can invade repressive H3K27me3-marked domains with consequent H3K27me3 reduction (Figure 3P). Moreover, the data suggest that the ESC + 2i-specific role of BAZ2A to regulate H3K27me3 occupancy might depend by the specific chromatin structure of ESC + 2i that forms H3K27me3 bodies, which are absent in ESC + serum.

### BAZ2A bodies sequester BAZ2A to maintain gene repression at H3K27me3-marked chromatin

**Figure 4:**
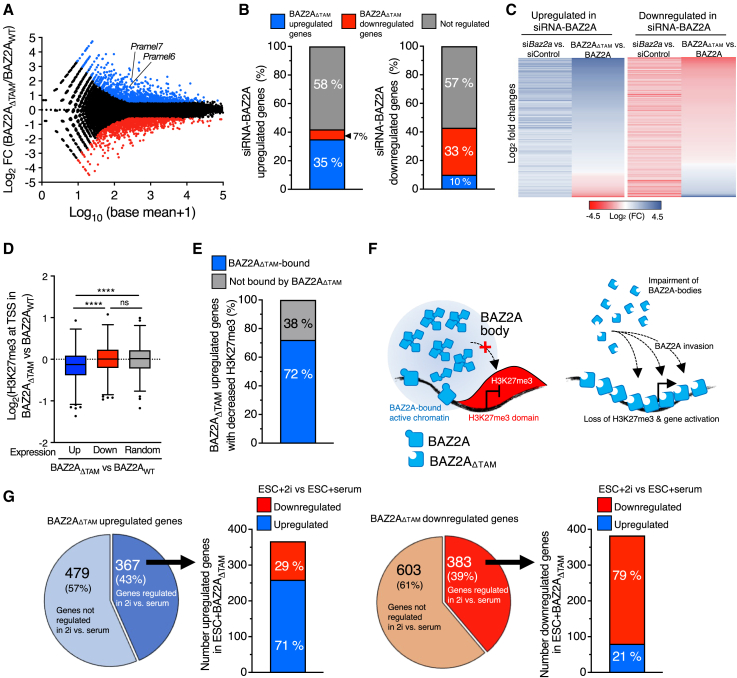
BAZ2A-TAM domain regulates gene expression (A) Volcano plot showing log_2_ fold change transcript levels of ESC + F/H-BAZ2Ar_ΔTAM_ vs. ESC + F/H-BAZ2Ar_WT_. (B) Proportion of BAZ2A-regulated genes significantly regulated in ESC + BAZ2A_ΔTAM_. (C) Heatmap showing fold changes of upregulated and downregulated transcripts in ESC + *Baz2a*-siRNA and corresponding fold changes in ESC + F/H-BAZ2Ar_ΔTAM_ vs. ESC + F/H-BAZ2Ar_WT_. (D) Fold changes of H3K27me3 levels in ESC + F/H-BAZ2Ar_ΔTAM_ vs. ESC + F/H-BAZ2Ar_WT_ at genes up- or downregulates in ESC + BAZ2Ar_ΔTAM_. Error bars represent SD. Statistical significance was calculated with unpaired two-tailed t test (^∗∗∗∗^p < 0.0001; ns, nonsignificant). (E) Proportion of genes upregulated and bound by BAZ2A_ΔTAM_ with decreased H3K27me3 levels in ESC + F/H-BAZ2Ar_ΔTAM_. (F) Model showing how the lack of the BAZ2A-TAM domain impairs body formation and promotes BAZ2A invasion into H3K27me3 domains, the loss of H3K27me3, and upregulation of gene expression. (G) Quantifications of up- and downregulated genes in ESC + F/H-BAZ2Ar_ΔTAM_ significantly up- or downregulated in ESC + 2i relative to ESC + serum.

To determine whether BAZ2A bodies in ESCs are required for BAZ2A-mediated gene regulation, we performed RNA sequencing (RNA-seq) of ESC + F/H-BAZ2Ar_WT_ and ESC + F/H-BAZ2Ar_ΔTAM_. BAZ2A_ΔTAM_ significantly altered the expression of 1,832 genes (BAZ2A-TAM regulated genes log_2_ fold change ≥ 0.58; p < 0.05), of which 846 genes were upregulated and 986 were downregulated compared with ESC + F/H-BAZ2Ar_WT_ (Figure 4A; Table S2). We intersected BAZ2A-regulated genes identified in a previous RNA-seq analysis of BAZ2A-depleted ESC + 2i^24^ with genes differentially expressed upon BAZ2A_ΔTAM_ expression. 35% of genes upregulated upon BAZ2A-KD showed the same expression changes in ESC + F/H-BAZ2Ar_ΔTAM_, whereas only 7% were downregulated (Figures 4B and 4C). We obtained a similar trend for downregulated genes, indicating that a large fraction of BAZ2A-regulated genes depends on BAZ2A-TAM domain. Gene ontology terms of BAZ2A_ΔTAM_-regulated genes showed significant enrichment in pathways linked to development (Figure S5A; Table S3). Interestingly, genes upregulated in ESC + BAZ2A_ΔTAM_ significantly decreased H3K27me3 levels relative to random genes or genes downregulated by ESC + BAZ2A_ΔTAM_, and a large fraction of these genes (72%) were bound by BAZ2A_ΔTAM_, indicating a direct role of BAZ2A in the reduction of H3K27me3 (Figures 4D–4F). These results further suggest that BAZ2A bodies sequester BAZ2A to maintain gene repression at H3K27me3-marked chromatin.

BAZ2A_ΔTAM_ did not affect pluripotency since the expression of pluripotency genes was not altered. However, BAZ2A_ΔTAM_ enhanced the ground-state gene signature of ESC + 2i (Figure 4G). 43% of BAZ2A_ΔTAM_-upregulated genes were differentially expressed in ESC + 2i vs. ESC + serum, and 72% of them were significantly upregulated in ESC + 2i. We obtained similar results for downregulated genes. Among genes upregulated in both ESC + BAZ2A_ΔTAM_ and ESC + 2i, *Pramel6* and *Pramel7*, which were implicated in ground-state pluripotency,^28^^,^^29^ showed a significant upregulation in ESC + BAZ2A_ΔTAM_ (Figures 4A and S5B). Since BAZ2A-body loss decreased repressive chromatin states and enhanced ground-state transcriptional signature, we asked whether ESC + BAZ2A_ΔTAM_ could differentiate faster. After induction of monolayer differentiation upon withdrawal of LIF, *Nestin* (a marker of neuroectoderm lineage) and *Bmp4* (mesoderm) were significantly higher expressed in differentiated ESC + BAZ2A_ΔTAM_ relative to control cells, whereas *Fgf5* (endoderm) and pluripotency genes were not affected (Figure S5C). These results suggest that ESC + BAZ2A_ΔTAM_ are in a more plastic state and can activate differentiation genes faster.

### BAZ2A bodies depend on active transcription

**Figure 5:**
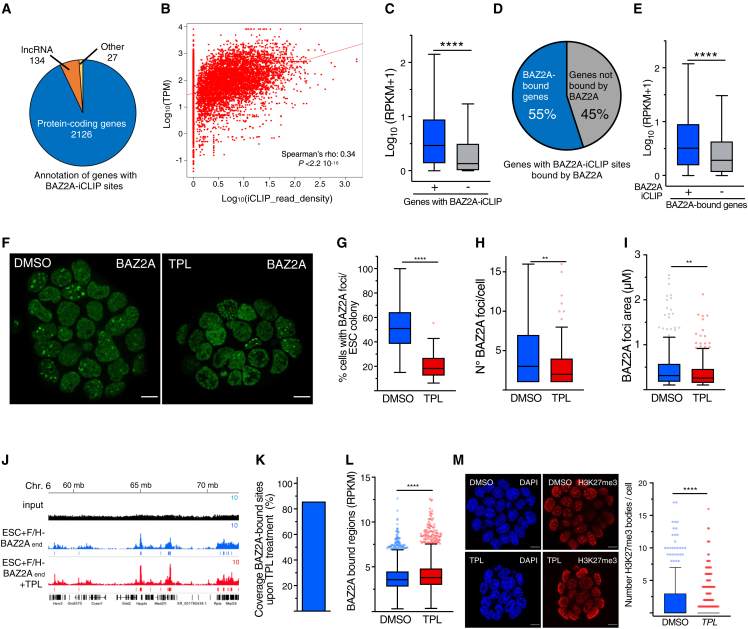
BAZ2A associates with RNA (A) Pie chart showing genome annotation of BAZ2A-iCLIP sites. (B) Scatterplot showing the correlation between gene expression and BAZ2A-iCLIP read density. (C) Boxplot showing the expression level of genes containing or depleted of BAZ2A-iCLIP sites. Values are shown as log_10_ average RPKM. (D) Pie chart showing the percentage of BAZ2A-bound genes containing BAZ2A-iCLIP sites among all genes with BAZ2A-iCLIP sites. (E) Boxplot showing expression level of BAZ2A-bound genes containing or depleted of BAZ2A-iCLIP sites. Values are shown as log_10_ average RPKM. (F) Representative live-cell images showing ESC + mGFP-BAZ2A_end_ treated with triptolide (TPL) for 4 h. Scale bars: 10 μm. (G–I) Boxplots showing amounts of cells with BAZ2A bodies (G) and the number (H) and area (I) of BAZ2A bodies in ESCs treated with TPL. (J) Tracks of BAZ2A-ChIP-seq displaying BAZ2A occupancy in ESCs treated with TPL. (K) Bar diagram showing the coverage of BAZ2A-bound sites in ESCs treated with TPL. (L) Boxplots showing the levels of BAZ2A occupancy in ESCs treated with DMSO or TPL for 4 h. (M) Representative immunofluorescence images showing H3K27me3 in ESC treated with triptolide (TPL) for 4 h. Scale bar represents 10 μm. Quantifications of the number of H3K27me3 bodies/cell are shown. Statistical significance in boxplots in (C), (E), (G)–(I), (L), and (M) was calculated with Mann-Whitney test (^∗∗^p < 0.01 ^∗∗∗∗^p < 0.0001).

Since BAZ2A bodies depend on RNA, we performed individual-nucleotide resolution UV-crosslinking and immunoprecipitation (iCLIP) to identify BAZ2A-interacting RNAs in ESCs expressing endogenous F/H-BAZ2A (F/H-BAZ2A_end_)^24^ (Figure S6A). F/H-BAZ2A immunoprecipitates from UV-crosslinked cells showed a stronger radioactive signal than in uncrosslinked cells, suggesting a direct interaction of BAZ2A with RNA (Figure S6B). RNA labeling was sensitive to high Rnase I treatment, confirming that the material crosslinked to BAZ2A was RNA. We sequenced BAZ2A-iCLIP libraries from three independent biological experiments. BAZ2A-iCLIP peaks had a median length of 58 nt, and the majority of them overlapped with protein-coding genes and were enriched within the coding regions, including introns (Figures 5A, S6C, and S6D). Motif enrichment analysis identified several novel motifs and sequences recognized by ZNF524 and the splicing factor protein SRSF1 (Figure S6E). The interaction strength of BAZ2A with RNA (defined by iCLIP read density) was directly proportional to transcript levels, and genes whose transcripts are bound by BAZ2A were higher expressed than genes lacking BAZ2A-iCLIP sites (Figures 5B and 5C). A large fraction (55%) of RNAs containing BAZ2A-iCLIP sites derived from BAZ2A-bound genes, and these RNAs showed higher expression levels relative to RNA from BAZ2A-bound genes lacking BAZ2A-iCLIP sites (Figures 5H and 5I). These results suggest that BAZ2A associates with RNA originating from BAZ2A-bound genes. However, only a few BAZ2A-interacting RNAs corresponded to BAZ2A-regulated genes, indicating that this interaction is not directly implicated in gene regulation (Figure S6F).

To determine whether transcription affects BAZ2A-body and BAZ2A association with chromatin, we treated ESC + mGFP-BAZ2A_end_ for 4 h with RNA Pol II transcription inhibitor triptolide (TPL). This treatment did not affect *Baz2a* mRNA levels (Figure S6G). TPL treatment reduced the number of BAZ2A bodies, which also appear significantly smaller compared with control cells (Figures 5G–5I). We obtained similar results by inhibiting transcription with actinomycin D (Figure S6H). These results are also consistent with the impairment of BAZ2A bodies observed upon treatment with Rnase A (Figure 1C). Next, we asked whether 4-h treatment with TPL was sufficient to alter BAZ2A association with chromatin by performing quantitative FLAG-ChIP-seq analysis of ESC + F/H-BAZ2A_end_ treated with TPL (Figures 5J–5L). BAZ2A genome coverage was not affected, however, and similarly to BAZ2A_ΔTAM_, BAZ2A amounts bound to chromatin were higher in ESC + TPL than control cells (Figures 5K and 5L). These results indicate that active transcription is required for BAZ2A bodies, thereby limiting BAZ2A association to chromatin. Moreover, they suggest that BAZ2A interaction with RNAs originating from BAZ2A-bound genes might be important for BAZ2A bodies. Considering the PS features of BAZ2A bodies, BAZ2A interactions with these RNA should probably be weak and transient and RNA sequence independent.

Similarly to BAZ2A, transcription inhibition significantly decreased the number of H3K27me3 bodies (Figures 5M and S6H). Thus, loss of BAZ2A bodies upon transcription inhibition might impair H3K27me3 bodies, although we cannot exclude that H3K27me3 bodies can also directly depend on nascent transcription and, in general, on RNA, as previously reported.^30^

Together, the results indicate that BAZ2A associates with RNAs originating from BAZ2A-bound genes and that BAZ2A-body formation depends on active transcription and serves to limit BAZ2A invasion into chromatin.

### BAZ2A associates with *Malat1* and chromatin in contact with nuclear speckles

**Figure 6:**
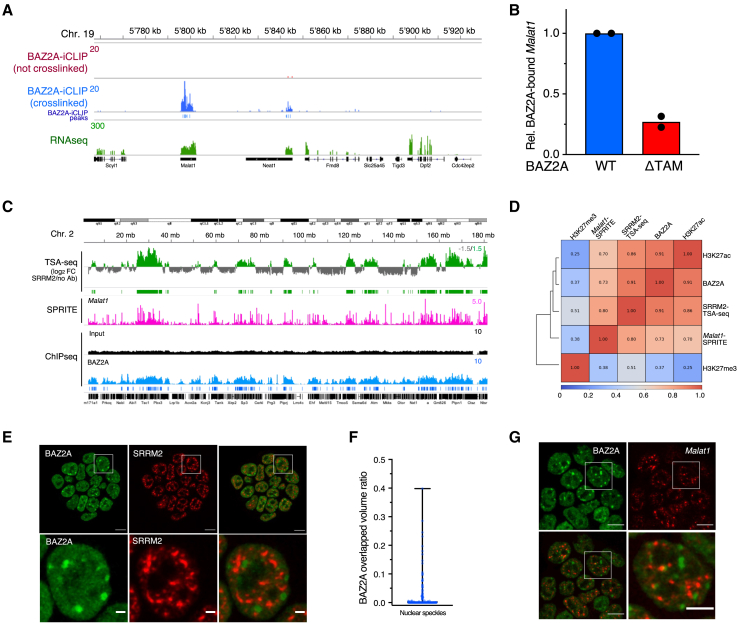
BAZ2A associates with *Malat1* and regulates nuclear speckles (A) Tracks showing *Malat1* signal in BAZ2A-iCLIP and RNA-seq. (B) Levels of *Malat1* from HA-RIP in ESC + F/H-BAZ2Ar_WT_ and ESC + F/H-BAZ2Ar_ΔTAM_. Data are normalized to BAZ2A_WT_-bound *Malat1* and are from two independent experiments. (C) Tracks showing SRRM2-TSA-seq *Malat1*-association to chromatin from SPRITE^33^ and BAZ2A-ChIP-seq. (D) Spearman’s correlation heatmap of BAZ2A, H3K27me3, and H3K27ac ChIP-seq, SRRM2 TSA-seq, and *Malat1* SPRITE.^33^ BAZ2A ChIP-seq and SRRM2-TSA-seq are from this work. H3K27ac and H3K27me3 ChIP-seq and *Malat1* SPRITE were from Dalcher et al.^24^ and Quinodoz et al.^33^ (E) Representative immunofluorescence images showing BAZ2A and SRRM2-marked nuclear speckles. Scale bars, top images: 5 μm; bottom images: 2 μm. (F) Quantification of overlapping volume of BAZ2A bodies over nuclear speckles. Data are from 25 cells and 184 BAZ2A bodies. (G) Representative immuno-RNA-FISH images of BAZ2A and *Malat1*. Scale bar is 10 μm, magnified image 5 μm.

Although the results shown so far suggested a promiscuous association of BAZ2A with nascent RNAs, the lncRNA metastasis-associated lung adenocarcinoma transcript 1 (*Malat1*)^31^ was one of the top BAZ2A-interacting RNAs (Figure 6A; Table S4). We reasoned that high expression levels of *Malat1* might not probably be the only reason for the detection of high BAZ2A-iCLIP signals since not all highly expressed RNAs interact with BAZ2A. We validated BAZ2A-*Malat1* association by RNA IP (RIP), which also showed that the interaction with *Malat1* depends on BAZ2A-TAM domain (Figure 6B). *Malat1* predominantly localizes at the periphery of nuclear speckles, which are surrounded by active chromatin domains.^31^^,^^32^ Accordingly, recent SPRITE analyses showed that *Malat1* associates with active chromatin regions.^33^ To determine whether BAZ2A-*Malat1* interaction might be indicative of the proximity of BAZ2A-bound chromatin to nuclear speckles, we performed tyramide signal amplification TSA-seq^34^ using antibodies against the nuclear speckles SRRM2 protein^35^^,^^36^ (Figure 6C). Remarkably, BAZ2A-bound regions highly correlated with regions mapped in proximity to nuclear speckles by SRRM2-TSA-seq and defined to interact with *Malat1* by SPRITE, indicating that BAZ2A-bound chromatin can associate with nuclear speckles (Figures 6C and 6D). Surprisingly, Ifs for BAZ2A and SRRM2 revealed that BAZ2A bodies rarely contact nuclear speckles and show very little overlapped volume (Figures 6E and 6F). We had a similar observation for *Malat1*-marked nuclear speckles using RNA-FISH analyses (Figure 6G). Thus, the data suggested the presence of two populations of BAZ2A-bound chromatin, one within BAZ2A bodies that is close to H3K27me3 domains and the other one in proximity of nuclear speckles that is not within BAZ2A bodies.

### *Malat1* regulates BAZ2A association to chromatin and limits BAZ2A-body formation

**Figure 7:**
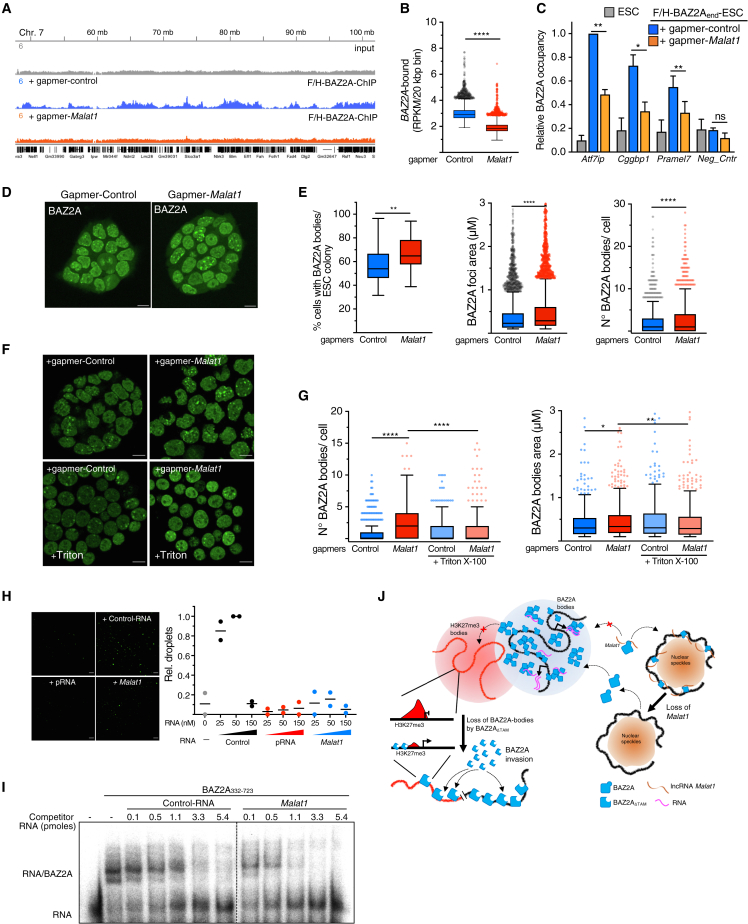
*Malat1* regulates BAZ2A association to chromatin and limits body formation (A) Tracks showing BAZ2A-ChIP-seq profile in ESC + gapmer-control or gapmer-*Malat1*. (B) BAZ2A levels at BAZ2A-bound regions in ESC control and corresponding levels in ESC + gapmer-*Malat1*. Values are shown as average RPKM. Statistical significance was calculated using the paired two-tailed t test (^∗∗∗∗^p < 0.0001). (C) Anti-FLAG ChIP-qPCR of ESCs and ESC + F/H-BAZ2A_WT_ treated with gapmer-control or gapmer-*Malat1*. Data were normalized to input and to *Atf7ip*. Average values of three independent experiments. Error bars represent SD, and statistical significance was calculated using the paired two-tailed t test (^∗^p < 0.05, ^∗∗^p < 0.01; ns, nonsignificant). (D) Representative images showing ESC + mGFP-BAZ2A_end_ treated with gapmer-control or gapmer-*Malat1*. Scale bars: 10 μm. (E) Proportion of cells with BAZ2A bodies and area and number of BAZ2A bodies/cells in ESC + gapmer-control or gapmer-*Malat1*. (F) Representative images of ESC + mGFP-BAZ2A_end_. When indicated, cells were treated with Triton X-100 prior fixation. Scale bars: 10 μm. (G) Quantification of area and number of BAZ2A bodies/cells in ESCs. (H) Representative images of droplets using 500 nM recombinant mGFP-BAZ2A_N_, in the absence or presence of 50 nM RNA-control, pRNA, or *Malat1*. Scale bars: 2 μm. Right panel, quantifications of the number of droplets under the indicated RNA concentrations. Values are from two independent experiments. (I) BAZ2A binds to *Malat1*. Increasing equal moles of *in vitro* transcripts corresponding to RNA-Control and *Malat1* sequences were used to compete for binding of recombinant BAZ2A_332–723_ to radiolabeled control RNA. RNA/protein complexes were analyzed by EMSA. (J) Model showing the role of BAZ2A condensates and *Malat1* in chromatin regulation of ESCs. BAZ2A condensates are close to H3K27me3 bodies and depend on BAZ2A-TAM domain and active transcription. BAZ2A bodies sequester BAZ2A and limit BAZ2A invasion into chromatin. The model shows how BAZ2A_ΔTAM_ occupies H3K27me3 chromatin with consequent H3K27me3 reduction and activation of gene expression. On the right, BAZ2A associates with *Malat1* and chromatin contacting nuclear speckles. *Malat1* is required for BAZ2A binding to chromatin and counteracts BAZ2A body formation, which do not contact nuclear speckles. Statistical significance in boxplots in (E) and (G) was calculated with Mann-Whitney test (^∗^p < 0.05, ^∗∗^p < 0.01, ^∗∗∗∗^p < 0.0001; ns, nonsignificant). Error bars represent SD.

To determine whether BAZ2A binding to chromatin depends on *Malat1*, we performed FLAG-ChIP-seq in F/H-BAZ2A_end_-ESCs depleted of *Malat1*. We found that BAZ2A association with chromatin was impaired (Figures 7A, 7B, and S7A). We validated these results by ChIP-qPCR by measuring BAZ2A association at known BAZ2A-bound genes (Figure 7C). This result differed from the TPL-mediated transcription inhibition that causes impairment of BAZ2A bodies while promoting BAZ2A association to chromatin (Figure 5), suggesting a distinct role of *Malat1* for BAZ2A interaction with chromatin. Accordingly, 4 h treatment with TPL did not alter *Malat1* levels (Figure S7B). *Malat1*-KD caused a minor downregulation of BAZ2A mRNA and protein levels (ca. 25% reduction), suggesting that *Malat1* can regulate BAZ2A expression and *Malat1*-KD might partially contribute to the reduced BAZ2A chromatin association (Figures S7C and S7D). To determine whether loss of BAZ2A binding to chromatin upon *Malat1*-KD affects BAZ2A bodies, we performed Ifs in *Malat1*-depleted ESCs (Figures 7D and 7E). *Malat1*-KD induced a significant increase in the area and number of BAZ2A bodies. We reasoned that this enlargement could be caused by an increased pool of free BAZ2A moieties (i.e., not bound to chromatin), which can form bodies. To test this, we measured BAZ2A bodies in *Malat1*-KD ESCs upon extraction of proteins not bound to chromatin using Triton X-100 prior imaging (Figures 7F and 7G). The enlarged BAZ2A bodies observed in *Malat1*-KD ESCs significantly decreased their size and number when treated with Triton X-100, suggesting that the lack of *Malat1* releases BAZ2A from chromatin, making it available for self-assembly and bodies formation. The data also suggested that *Malat1* through its association with BAZ2A-TAM domain should negatively regulate BAZ2A-body formation, explaining also why BAZ2A bodies do not contact nuclear speckles. Thus, we measured *in vitro* mGFP-BAZ2A_N_-droplet formation in the presence of diverse RNAs of similar size and different concentrations, using sub-conditions for the formation of BAZ2A droplets in the absence of RNA (500 nm BAZ2A and 2% PEG8000) (Figure 7H). We used pRNA (−220 to −1 of rRNA genes), the lncRNA known to strongly interact with BAZ2A-TAM domain in differentiated cells.^19^^,^^20^ As control, we used RNA sequences (control RNA), which were shown to have lower binding affinity to BAZ2A than pRNA.^19^^,^^22^ For *Malat1*, we used sequences containing BAZ2A-iCLIP signals (+4,868 to +5,065). Electrophoretic mobility shift competition assay (EMSA) revealed that this *Malat1* region shows higher binding affinity to BAZ2A-TAM than control RNA (Figure 7I). In the presence of 25 and 50 nM control RNA, the number of BAZ2A droplets increased compared with droplets formed in the absence of RNA, indicating that RNA with weak BAZ2A binding promotes BAZ2A-body formation. The effect of RNA in promoting BAZ2A droplets depended on BAZ2A-TAM domain since BAZ2A_ΔTAM_ could not form droplets in presence of RNA (Figure S7F). The data also showed that elevated RNA concentrations (150 nM) impaired droplet formation, a result consistent with previous work showing that the negative charges exceed at high RNA concentration and the repulsion between the charges causes the dissociation of PS.^18^ Remarkably, both strong BAZ2A-interacting RNAs, pRNA or *Malat1*, impaired droplet formation. These results indicate that the nature of RNA interaction affects the formation of BAZ2A condensates. RNA with low binding affinity to BAZ2A promotes PS properties for BAZ2A-body formation, explaining also why BAZ2A bodies in cells depend on RNA and active transcription. On the other hand, strong BAZ2A-interacting RNAs, such as pRNA and *Malat1*, both mediating stable BAZ2A association to chromatin, impair BAZ2A condensates, most likely by blocking the TAM domain that is required for BAZ2A self-assembly (Figure 7J).

## Discussion

The classical view for the presence of diverse nuclear compartments is that they serve to locally establish high concentrations of factors, allowing a much greater efficiency of specific reactions.^37^ In this work, we have provided an additional layer of function and regulation by showing that BAZ2A, which binds active chromatin, gets sequestered into bodies to limit its invasion into adjacent H3K27me3 chromatin compartments, thereby preserving repressive chromatin and gene expression states (Figure 7J). BAZ2A-body formation is mediated by weak interactions between BAZ2A-TAM domain and nascent transcripts but negatively regulated by the strong interaction with the lncRNA *Malat1*, which in turn is required for BAZ2A association with active chromatin contacting the nuclear speckles.

IDRs are key protein domains in condensates.^12^^,^^13^^,^^14^ Although BAZ2A is predicted to contain some IDRs^38^^,^^39^ (Figures S7F and S7G), the driving force of BAZ2A condensates is the non-disordered TAM domain, suggesting that nuclear bodies formation can also be mediated by a structured RNA-binding domain. However, in this case, it appears that the binding strength to RNA is relevant to promote or impair condensate formation. The dependency of BAZ2A bodies on RNA and active transcription suggests that transient or weak BAZ2A-TAM interactions with transcripts originating from BAZ2A-bound regions are key for condensate formation. These results are also consistent with previous works showing condensate formation at site of transcription^12^^,^^40^^,^^41^ and suggesting that the role of RNA in transcriptional condensates is likely to be driven by electrostatic interactions and be sequence independent.^18^^,^^42^ Accordingly, BAZ2A droplets are promoted by RNA sequences that show weaker binding affinity to BAZ2A-TAM domain than the lncRNA pRNA^19^^,^^22^ and *Malat1*, which both impair BAZ2A droplets. Thus, the different binding affinities of RNAs with BAZ2A can have profound effects on BAZ2A bodies. Accordingly, in ESCs, *Malat1* acts as a negative regulator of BAZ2A condensates while promoting BAZ2A association with chromatin, a result further supported by the lack of BAZ2A bodies contacting nuclear speckles where *Malat1* is mainly localized. It has been reported that pRNA contains a stem-loop structure that mediates the interaction with BAZ2A.^22^^,^^43^ Recent studies showed that BAZ2A-TAM domain preferentially associates with double-strand RNA, a structure mirroring the RNA stem-loop.^44^ Interestingly, the murine *Malat1* sequence interacting with BAZ2A shares 86% identity with human *MALAT1*, which contains a stem-loop structure present in 53 vertebrate *Malat1* homologs,^45^ suggesting that BAZ2A can interact with *Malat1* using features similar to pRNA. Thus, *Malat1* has a dual function in ESCs: on one side, it impairs BAZ2A bodies, and on the other side, it promotes BAZ2A association with active chromatin that often contacts nuclear speckles. This different dependency of BAZ2A bodies on RNA is consistent with previous works suggesting that different RNAs can impart distinct biophysical properties of droplets. For example, different mRNAs can affect droplets formed by WHI3, a factor characterized by large IDRs, by altering viscosity propensity to fuse and exchange rates of components with bulk solution.^46^ Recently, it has been reported that DHH1 condensates lose droplet fluidity and acquired an irregular shape in the presence of structured RNA compared with the unstructured poly(U) RNA.^47^ Similarly proline/arginine-rich peptides can form liquid-like condensates with poly(A), poly(U), and poly(C) RNA, whereas poly(G)RNA forms gel-like networks.^48^ Compared with these examples, however, the role of pRNA or *Malat1* in BAZ2A droplet formation seems to be mechanistically different since they impair droplet formation through their strong association with the structured TAM domain.

Our previous studies have analyzed BAZ2A in several cell types, but in none of them, BAZ2A bodies were detectable.^19^^,^^22^^,^^24^^,^^49^^,^^50^ Instead, and in contrast to ESCs, BAZ2A was largely enriched in the nucleolus, where it acts as the main chromatin repressor of rRNA genes through the interaction with pRNA. BAZ2A is expressed at much higher levels in ESCs compared with differentiated cells^24^ and appears to be one of the most abundant chromatin-bound proteins in ESCs.^51^ Thus, and in analogy to the dependency of condensates on protein concentration,^5^^,^^52^ the high abundance of BAZ2A could also be one of the factors promoting the formation of BAZ2A bodies specifically in ESCs.

The presence of H3K27me3 bodies in ESC + 2i highlighted an additional difference between ESC + 2i and ESC + serum and should explain why BAZ2A can regulate H3K27me3 only in ESC + 2i. Indeed, in the linear genome, BAZ2A does not associate with H3K27me3-marked chromatin; however, in the nuclear space, BAZ2A bodies and H3K27me3 bodies frequently overlap. What are these H3K27me3 bodies? Considering that H3K27me3 is incorporated into chromatin, these bodies could represent highly structured or interacting chromatin domains. The fact that they uniquely form in ESC + 2i and depend on RNA and active transcription paves the way for future studies.

The inability of BAZ2A_ΔTAM_ mutant to form bodies and its increased association with chromatin suggest that BAZ2A bodies serve to sequester unbound BAZ2A, thereby limiting its invasion into proximal chromatin domains, including H3K27me3-marked chromatin. Thus, the loss of BAZ2A bodies should release a certain amount of BAZ2A that can compete for Polycomb binding and modify H3K27me3-marked chromatin. This action could probably be facilitated either by the release of regulators of active chromatin such as BRD3 and BRD4, which can partition into BAZ2A bodies, or by active chromatin regulators known to interact with BAZ2A.^24^

Collectively, our data have shown a direct communication between distinct nuclear compartments based on PS mechanisms that are regulated according to the strength of RNA interactions with a structured RNA-binding proteins and serve to regulate chromatin and gene expression states.

### Limitations of the study

Our study has demonstrated a direct and functional connection between active and repressive nuclear compartments through PS mechanisms that serve to sequester BAZ2A and safeguard repressive chromatin domains. The microscopy data have shown that about 50% of ESCs display large BAZ2A and H3K27me3 bodies. This phenotype could be attributed to heterogeneity between ESCs. However, we cannot exclude the possibility that cells that do not display large bodies may still contain small BAZ2A bodies that are not detectable by standard confocal microscopy but have similar biophysical properties and exert similar functions as large BAZ2A and H3K27me3 bodies. The genomic data (RNA-seq, ChIP-seq, etc.) cannot distinguish between ESCs with large or no/undetectable bodies. Considering also that ESC + BAZ2A_ΔTAM_ altered H3K27me3 chromatin distribution with a minimal effect on global H3K27me3 levels, image analyses can only distinguish loss of H3K27me3 bodies but not altered H3K27me3 organization between cells with or without large BAZ2A and H3K27me3 bodies. Finally, although we discovered the ability of BAZ2A to occupy and modify repressive chromatin upon release from bodies, the exact mechanism by which BAZ2A competes with Polycomb for chromatin binding and the order of events to lose repressive features remain yet to be defined.

## STAR★Methods

### Key resources table

**Table:** 

REAGENT or RESOURCE	SOURCE	IDENTIFIER
**Antibodies**		

Rabbit polyclonal anti-HA	Abcam	Cat# ab9110; RRID: AB_307019
Rabbit polyclonal anti-GFP	Abcam	Cat# ab290; RRID: AB_303395
Mouse monoclonal anti-SNF2H	Santa Cruz Biotechnology	Cat# sc-365727; RRID: AB_10844618
Rabbit polyclonal anti-H327me3	Active Motif	Cat# 39155; RRID: AB_2561020
Rabbit polyclonal anti-H3	Abcam	Cat# ab1791; RRID: AB_302613
Mouse monoclonal anti-GST	Santa Cruz Biotechnology	Cat# sc-138; RRID: AB_627677
IRDye® 800CW Goat anti-Mouse IgG Secondary Antibody	Li-cor	Cat# 926-32210; RRID: AB_621842
IRDye® 680RD Goat anti-Mouse IgG Secondary Antibody	Li-cor	Cat# 926-68070; RRID: AB_10956588
IRDye® 800CW Goat anti-Rabbit IgG Secondary Antibody	Li-cor	Cat# 926-32211; RRID: AB_621843
IRDye® 680RD Goat anti-Rabbit IgG Secondary Antibody	Li-cor	Cat# 926-68071; RRID: AB_10956166
Mouse monoclonal anti-B23 (NPM1)	Sigma-Aldrich	Cat# B0556; RRID: AB_2154872
Rabbit polyclonal anti-SRRM2	Novus Biologicals	Cat# NBP2-55697
Mouse monoclonal anti-SC35	Abcam	Cat# ab11826; RRID: AB_298608
Goat anti-Rabbit IgG (H+L) Highly Cross-Adsorbed Secondary Antibody Alexa Fluor 488	Invitrogen	Cat# A11034; RRID: AB_2576217
Goat anti-Rabbit IgG (H+L) Highly Cross-Adsorbed Secondary Antibody Alexa Fluor 546	Invitrogen	Cat# A11035; RRID: AB_2534093
Goat anti-Mouse IgG (H+L) Highly Cross-Adsorbed Secondary Antibody Alexa Fluor 546	Invitrogen	Cat# A11030; RRID: AB_2737024
Goat anti-Rabbit IgG (H+L) Highly Cross-Adsorbed Secondary Antibody Alexa Fluor 647	Invitrogen	Cat# A21245; RRID: AB_2535813
Anti-H2Av (D. Melanogaster)	Active Motif	Cat# 61686; RRID: AB_2737370

**Bacterial and virus strains**		

DH5α competent cells	Invitrogen	18265017
BL21(DE3) Competent Cells	Invitrogen	EC0114

**Chemicals, peptides, and recombinant proteins**		

Amicon Ultra-0.5 Centrifugal Filter Unit	Millipore	UFC5050
Bovine Serum Albumin	Sigma-Aldrich	A2153
CHIR99021	Sigma	SML1046
cOmplete™ Protease Inhibitor Cocktail	Roche	11697498001
Costar® Spin-X® Centrifuge Tube Filters	Corning	8161
Diisopropylfluorophosphate	Sigma-Aldrich	D0879-1G
DMEM-F12	Gibco	21041025
DNase I	Thermo Scientific™	EN0525
Dynabeads™ Protein A	Invitrogen	10002D
Dynabeads™ Protein A	Invitrogen	10002D
Formaldehyde	Sigma-Aldrich	47608
Formamide	Sigma-Aldrich	F9037
Glutathione Sepharose® 4B beads	Cytiva	17-0756-01
HisTrap™ High Performance column	Cytiva	17-5247-01
LIF	Polygene	N/A
Matrigel	Corning	356238
MNase	Roche	10107921001
NEBNext® Ultra™ II DNA Library Prep for Illumina	New England Biolabs	E7645S and E7645L
Neurobasal™ Medium	Gibco	12348017
PD0325901	Sigma	391210-10-9
PreScission Protease	GenScript	Z02799
Proteinase K	Thermo Fisher Scientific	EO0492
RNase A	Thermo Scientific™	EN0531
Slide-A-Lyzer cassette G1	Thermo	66380
T7 RNA polymerase	Thermo Fisher	EP0111
TritonX-100	Sigma-Aldrich	T8787
TRIzol reagent	Life Technologies	15596026
Tween 20	Merck	P9416
Tyramide-biotin	Sigma-Aldrich	SML2135
VECTASHIELD® Antifade Mounting Medium with DAPI	Vector Laboratories	H-1200-10
BAZ2A_N_-Hist	This work	N/A
BAZ2A_N/ΔTAM_-Hist	This work	N/A
BAZ2A_N_-mCherry-Hist	This work	N/A
BAZ2A_N_-mGFP-Hist	This work	N/A
BAZ2A_N/ΔTAM_-mGFP-Hist	This work	N/A

**Deposited data**		

RNAseq	This work	GEO: GSE225898
ChIPseq	This work	GEO: GSE225898
iCLIP	This work	GEO: GSE225898
TSAeq	This work	GEO: GSE225898

**Experimental models: Cell lines**		

One hundred and twenty-nine mouse embryonic stem cells (E14 line)	Savić et al.^22^	N/A
HEK293T	ATCC	N/A
ESC+mGFP-BAZ2A_end_	This paper	N/A
ESC+mGFP-BAZ2A_WT_	This paper	N/A
ESC+mGFP-BAZ2A_ΔTAM_	This paper	N/A
ESC+F/H-BAZ2Ar_WT_	This paper	N/A
ESC+F/H-BAZ2Ar_ΔTAM_	This paper	N/A
ESC+F/H-BAZ2A_end_	Dalcher et al.^24^	N/A

**Oligonucleotides**		

See Table S5	This paper	N/A

**Recombinant DNA**		

Super PiggyBac Transposase Expression Vector	SBI System Biosciences	PB210PA-1
ES-FUCCI plasmid	Addgene	62451
pSpCas9(BB)-2A-GFP	Addgene	48138
mGFP-BAZ2Ar	This paper	N/A
mGFP-BAZ2A_ΔTAM_	This paper	N/A
mGFP-BAZ2A_W551G/Y552A_	This paper	N/A
mGFP-BAZ2A_N_	This paper	N/A
F/H-BAZ2A_N_	This paper	N/A
mGFP-BAZ2A_C_	This paper	N/A
F/H-BAZ2A_C_	This paper	N/A
GST-BAZ2A_N_-Hist	This paper	N/A
GST-BAZ2A_N/ΔTAM_-Hist	This paper	N/A
GST-Hist-BAZ2A_N_	This paper	N/A
GST-Hist-BAZ2A_N/ΔTAM_	This paper	N/A
GST-BAZ2A_N_-mGFP-Hist	This paper	N/A
GST-BAZ2A_N_-mCherry-Hist	This paper	N/A
GST-BAZ2A_N/ΔTAM_-mGFP-Hist	This paper	N/A
Topo2.1-pRNA	Savić et al.^22^	N/A
Topo2.1-Control-RNA	Savić et al.^22^	N/A
Topo2.1-Malat1	This paper	N/A

**Software and algorithms**		

R 3.4.3	N/A	https://cran-archive.r-project.org/bin/windows/base/old/3.4.3/
bowtie2 2.2.5	N/A	https://sourceforge.net/projects/bowtie-bio/files/bowtie2/2.2.5/
samtools 1.7	N/A	https://sourceforge.net/projects/samtools/files/samtools/1.7/
deepTools 3.5.0	N/A	https://deeptools.readthedocs.io/en/3.5.0/content/advanced_features.html
bedtools 2.24.0	N/A	https://launchpad.net/ubuntu/+source/bedtools/2.24.0-1
SICER 1.1	N/A	https://personal.broadinstitute.org/anshul/projects/encode/preprocessing/peakcalling/sicer/SICER/README.pdf
MACS2 2.2.7.1	N/A	https://hub.docker.com/layers/fooliu/macs2/version-2.2.7.1
IGV 2.15.14	N/A	https://igv.org
Fiji 2.9.0/1.53t	N/A	https://imagej.net/software/fiji/
Prism GraphPad 10.1.0	GraphPad Software	https://www.graphpad.com/features
TOMTOM	N/A	https://meme-suite.org/meme/tools/tomtom
Imaris 9.8	Oxford Instruments	https://imaris.oxinst.com/versions/9-8

**Other**		

ANTI-FLAG® M2 Affinity Gel	Sigma-Aldrich	A2220
Pierce™ Anti-HA Magnetic Beads	ThermoFisher	88837
GFP-Trap® Magnetic Agarose	Chromotek	gtma
Pierce™ Anti-HA Magnetic Beads	ThermoFisher	88837
TransIT-X2 Dynamic Delivery System	Mirus Bio	MIR6004
Lipofectamine™ 2000 Transfection Reagent	Invitrogen	11668019
Malat1 Stellaris® FISH Probes with Quasar® 570 Dye	Biosearch technology	SMF-3008-1

### Resource availability

#### Lead contact

Further information and requests for resources and reagents should be directed to and will be fulfilled by the lead contact, Raffaella Santoro (raffaella.santoro@dmmd.uzh.ch).

#### Materials availability

All unique reagents generated in this study are available from the lead contact with a completed Materials Transfer Agreement.

#### Data and code availability

• The RNAseq, ChIPseq, iCLIP, and TSA-seq data generated in this study have been deposited in the NCBI’s GSE225898. These data are publicly available as of the date of publication.
• This paper does not report original code.
• Any additional information required to reanalyze the data reported in this paper is available from the lead contact upon request.

### Experimental model and study participant details

#### Cell lines and cell culture

One hundred and twenty-nine mouse embryonic stem cells (E14 line) were cultured in 2i medium composed of DMEM-F12 and Neurobasal medium (1:1, Life Technologies), supplemented with 1× N2/B27 (Life Technologies), 1× penicillin/streptomycin/l-glutamine (Life Technologies), 50 μM β-mercaptoethanol (Life Technologies), recombinant leukemia inhibitory factor, LIF (Polygene, 1,000 U/ml) and MEK and GSK3β inhibitors, 2i (Sigma CHIR99021 and PD0325901, 3 and 1 μM, respectively). ESCs were seeded at a density of 5x10^4^ cells/cm^2^ in culture dishes (Corning® CellBIND® surface) coated with 0.1% gelatin without feeder layer. Propagation of cells was carried out every 2 days using enzymatic cell dissociation.

To differentiate ESC, ESC+F/H-BAZ2Ar_WT_ and ESC+F/H-BAZ2Ar_ΔTAM_ were transfected with siRNA-*Baz2a* and cultured in 2i medium for 1 day. ESCs were differentiated by culturing for 5 days in complete medium in the absence of LIF (DMEM, 10% FCS, 1 mM sodium piruvate (Sigma), 1× NEAA (Life Technologies), 1× penicillin/streptomycin/l-glutamine, 100 μM β-mercaptoethanol) on 0.1% gelatin coated culture dishes. During the 5-days differentiation, media was exchanged every 2 days. Cells were harvested for total RNA isolation before starting the differentiation and after 3 and 5 days.

To generate ESCs expressing endogenous BAZ2A tagged at the N-terminus with mGFP, the BAZ2A locus on exon 3 three base pairs upstream of the ATG start codon was targeted with a sgRNA guide sequence (GTCGTTTGCCTCCATTTCTGT) that was cloned into pSpCas9(BB)-2A-GFP,^53^ a gift from Feng Zhang (Addgene plasmid # 48138; http://n2t.net/addgene:48138; RRID:Addgene_48138). This plasmid was co-transfected in ESCs at a molar ratio of 1:2 with the HDR repair template plasmid containing the mEGFP sequence flanked by 1 kb homology arms by using TransIT-X2 transfection reagent (Mirus Bio) following the manufacturing instructions. 15x10^4^ wild-type ESCs were seed into gelatin-coated 6 cm plate, let to attach for 4 hour and then transfected. After 2 days, positively transfected cells were FACS sorted for GFP expression and were then further cultured for additional 3 days. Subsequently, ESCs were seeded for single cell clone isolation. The clones were first screened for mEGFP-BAZ2A expression by live cell imaging and then by SDS-PAGE followed by Western blotting.

To generate ESCs expressing mGFP-BAZ2Ar_WT_, mGFP-BAZ2Ar_ΔTAM_, F/H-BAZ2Ar_WT_, and F/H- BAZ2Ar_ΔTAM_, 7.5x10^4^ wild-type ESCs were seeded into gelatin-coated 6 cm plate and let to attach for 4 hours. Cells were then transfected with 1.25 μg of the plasmid containing both the BAZ2A transgene and the puromycin N-acetyl-transferase gene and 0.5 μl of Super PiggyBac Transposase Expression Vector (SBI System Biosciences, PB210PA-1) using 7.5 μl Lipofectamine™ 2000 Transfection Reagent (Invitrogen, 11668019). After 48 hours, 2 μg/μl of Puromycin (Life Technologies) was used to select positively transfected cells. After recover, ESCs were further treated with 1 μg/μl of puromycin for three days. Finally, ESCs were seeded for single cell clone isolation. The mGFP-BAZ2Ar ESCs clones were first screened for mEGFP-BAZ2A expression by live cell imaging. Both the mGFP-BAZ2Ar and the F/H-BAZ2Ar ESCs clones were ultimately screened by SDS-PAGE followed by Western blotting to select the clones with a comparable expression level between the transgene and the endogenous BAZ2A. A similar strategy was used to establish ESCs stably expressing an adapted version of fluorescent, ubiquitination-based cell cycle indicator (FUCCI) that was optimized to visualize cell cycle progression in ESCs.^54^ The ES-FUCCI plasmid was a gift from Pierre Neveu (a gift from Pierre Neveu (Addgene plasmid # 62451; http://n2t.net/addgene:62451; RRID:Addgene_62451) and was subcloned into the Super PiggyBac Transposase Expression Vector.

### Method details

#### Transfection of cells

For transient transfection of ESCs a DNA-lipid complex solution was prepared in Opti-MEM™ I Reduced Serum Medium (Gibco 11058021) with 2.5 μg of plasmids expressing mEGFP-BAZ2Ar 1.5 μg of a plasmid expressing an shRNA targeting the endogenous *Baz2a* and 7.5 μl Lipofectamine™ 2000 Transfection Reagent. This mix was used to transfect 7.5x10^5^ of wild-type ESCs in suspension rotating for 5 minutes at room temperature. After spinning them down to remove the transfection solution cells were resuspended in fresh medium and seeded into a Matrigel-coated 6 cm plate. 24 hours post-transfection cells were fixed and used for immunofluorescence.

For transient transfection of HEK 293T cells 1.5x10^6^ cells were plated in 10 cm plates and grow for 8 to 16 hours. Cells were then co-transfected with plasmids expressing BAZ2A tagged with mGFP and FLAG/HA sequences using BES-CaCl_2_ transfection method. After 48 hours the cells were collected for immunoprecipitation experiments.

For transfection using siRNA or locked nucleic acid (LNA)-gapmer 8 x10^4^ parental ESCs were seed into gelatin-coated or Matrigel-coated 6 cm plate. Cells were let to attach for 4 hour and then transfected with 2 μl of 40 μM siRNA stock or 0.4 μl of a 50 μM LNA-gapmer stock using Lipofectamine RNAiMAX Transfection Reagent (Invitrogen 13778150) following the manufacturing instructions. siRNA and gapmers sequences are listed in Table S5.

#### Immunofluorescence and RNA in situ hybridization

Cells were plated on coverslips pre-coated with Matrigel (Corning, 356238) for 2 hours at 37°C. After 48 to 72 hours, cells were fixed using 3.7% formaldehyde (Sigma-Aldrich, 47608) in PBS for 10 minutes at room temperature. After two washes in PBS, cells were permeabilized with 0.5% TritonX-100 (Sigma-Aldrich, T8787) in PBS for 10 minutes on ice and 5 minutes at room temperature. Cells were then washed two times in PBS and blocked with 1% Bovine Serum Albumin, (BSA, Sigma-Aldrich, A2153) in PBS-0.1% Tween 20 (Merck, P9416) for 1 hour at room temperature in a humidified chamber. The coverslips were then incubated with primary antibody resuspended in blocking solution overnight at 4°C in a humidified chamber. After 3 washes in PBS-0.1% Tween 20, the coverslips were incubated with secondary Alexa Fluor® antibody resuspended 1:1000 in Blocking Solution for 1 hour at room temperature. After 3 washes in PBS-0.1% Tween 20, the coverslips were mounted with VECTASHIELD® Antifade Mounting Medium with DAPI (H-1200-10), sealed with nail polish and imaged within one week.

For RNase A treatment, cells were treated prior fixation with 0.05% TritonX-100 in PBS for 30 seconds at room temperature, washed once with PBS, and incubated with 0.1 μg/μl RNase A (Thermo Scientific™, EN0531) in PBS for 10 minutes at room temperature. Cells were subsequentially fixed using 3.7% formaldehyde for 10 minutes at room temperature. After removing the fixation solution, cells were washed once with PBS and let sit upside down for 2 minutes. The coverslips were then permeabilized and processed for blocking and immunostaining as described above.

For the pre-extraction of not bound-chromatin proteins, cells were treated with 0.05% TritonX-100 in PBS for 3 or 10 minutes on ice. After washing with PBS, cells were fixed using 3.7% formaldehyde for 10 minutes at room temperature, washed again with PBS, and the coverslips were let sit upside down for 2 minutes. The coverslips were then directly processed for blocking and immunostaining as described above.

For RNA in situ hybridization, cells were prepared as mentioned above. After permeabilization, the coverslips were incubated in a humidified chamber overnight at 37°C in the hybridization solution (10% dextran sulphate (Millipore, S4030), 10% formamide (Sigma-Aldrich, F9037), 2x SSC (Saline Sodium Citrate), 0.02% BSA, 1 μg/μl yeast tRNA (Invitrogen, 15401-011) containing mouse Malat1 Stellaris® FISH Probes with Quasar® 570 Dye (biosearch technology, SMF-3008-1) diluted 1:100 from a 12.5 μM stock solution. Subsequently, the coverslips were washed twice in 2x SSC buffer containing 10% formamide for 30 minutes at 30°C and in 2x SCC and 1x SCC buffer for 15 minutes each at room temperature. The coverslips were mounted as described above.

#### Live cell imaging and FRAP

For live cell imaging, cells were plated in Matrigel-coated μ-Plate 96 or 24 well black plate (ibidi, 89626 and 82426) in 2i medium containing DMEM-F12 (Gibco, 21041025) and Neurobasal™ Medium, (Gibco, 12348017) without phenol red and imaged after 24-48 hours. 30 minutes before image acquisition, the imaging chamber was equilibrated to ensure controlled environmental conditions (5% CO_2_, 20% O_2_, 37°C, 95% humidity).

The FRAP experiments were performed by following the FRAP wizard in the Leica microscope. Briefly, a region of interest (ROI) corresponding to BAZ2A foci was bleached and images of the recovery were collected every 1.5 seconds. For the quantification, the fluorescence intensity of the ROIs was normalized to timepoint 0 and a non-bleached ROI within the same cell was used to normalize the recovery of the bleached ROI.

#### Image acquisition and analysis

Images for both fixed and live cells, were acquired with a Leica inverse SP8 FALCON (Fast Lifetime CONtrast) confocal laser scanning microscope, equipped with 63x HCX PL APO CS2 objective with oil immersion. For quantification a Z-stack (8-10 x 0.3 μm step size) of 10 to 15 colonies were imaged for each condition. The images processed using FIJI and analysed using ImageJ Macro Language (IJM) batch processing and Prism (Version 9.4.1).

For 3D analyses, serial images were acquired with a fixed voxel size of (x,y,z)=0.07,0.07,0.1 μm and constant excitation/emission settings throughout image replicates. Chromatic shifts were corrected following measurements on 500nm Spectra Beads. 3D images were segmented and quantified with Imaris 9.8 (Bitplane, Switzerland). BAZ2A, H3K27me3, and chromocenters were segmented as surface of adaptive size and background subtraction was applied following the Workflow 4b described in.^27^ Signal intensity, volumes and overlapped volume ratios were exported for plotting in Prism (Version 9.4.1).

#### Expression and purification of recombinant proteins

For protein expression, plasmids were transformed into BL21(DE3) cells and a bacterial colony was inoculated into Terrific Broth medium (TB, 24 g/L Yeast extract, 20g/L Tryptone, 4ml/L Glycerol, 0.017 M KH_2_PO_4_, 0.072 M K_2_HPO_4_) containing Ampicillin. A bacteria pre-culture was grown overnight at 37°C and 230 rpm and used to inoculate 6 liters of pre-warmed TB medium supplemented with Ampicillin in a dilution 1:100. The cells were shaken at 37°C and 230 rpm until they reached the log phase then were induced with 0.5 mM IPTG (Isopropil-β-D-1-tiogalattopiranoside) and grown for 4 more hours at 37°C and 130 rpm. Cells were spun down at 4°C and stored frozen at -80°C. Prior to lysate, the cells pellet was thawed on ice and resuspended in cold GST-Resuspension buffer (50 mM Tris-HCl pH 7.5, 750 mM KCl, 5% Glycerol, 1 mM DTT) freshly supplemented with a cocktail of protease inhibitor (Diisopropylfluorophosphate, Sigma-Aldrich, D0879-1G; cOmplete™ Protease Inhibitor Cocktail, Roche, 11697498001). Cells were lysed by 3 cycles of French press and incubated 30 minutes at 4°C with 1 μg/μl RNase A and 0.2 U/ml DNase I (Thermo Scientific™, EN0525). After spinning at 4°C for 30 minutes at 45’000g to remove insoluble particles, the lysate was incubated with Glutathione Sepharose® 4B beads (Cytiva, 17-0756-01) for 5 hours at 4°C, further supplemented with RNase and DNase. After 3 washes at 4°C for 20 minutes each, the protein solution was eluted by incubating the beads with cold GST-Elution buffer (50 mM Tris-HCl pH 8, 500 mM KCl, 5% Glycerol, 1 mM DTT, 10 mM Glutathione, cOmplete™ Protease Inhibitor Cocktail) freshly prepared, for 30 minutes at 4°C. The eluate was dialyzed overnight at 4°C with freshly made Dialysis buffer (50 mM Tris-HCl, 300 mM KCl, 5% Glycerol, 1 mM DTT, 7.5) supplemented with 2U/ml of PreScission Protease (GenScript, Z02799) to remove the GST tag. The eluate was filtered (0.22 μm) and loaded into a HisTrap™ High Performance column (Cytiva, 17-5247-01) using a ÄKTA go protein purification system. The column was washed (50 mM Tris-HCl pH 7.5, 1 M KCl, 5% Glycerol, 10 mM Imidazole, 1 mM DTT) and the protein was eluted with His-Elution buffer (50 mM Tris-HCl, 1 M KCl, 5% Glycerol, 1 mM DTT, pH 7.5) freshly prepared and supplemented with 500 mM Imidazole. After overnight dialysis at 4°C (50 mM Tris-HCl pH 7.5, 500 mM KCl, 5% Glycerol, 1 mM DTT), protein quality and amount were assessed by SDS-PAGE and Coomassie staining. Finally, single-use protein aliquots were snap frozen and stored at -80°C.

#### Droplet assay

Recombinant protein aliquots were thawed on ice and added to 50 mM Tris-HCl pH 7.5 buffer at different concentrations, with final KCl and PEG8000 concentrations as indicated in the figures. The protein solution was immediately loaded into a home-made chamber comprising a glass slide with a coverslip attached by two parallel strips of double-sided tape.^12^ Droplets were imaged with a Leica DMI6000 B inverted fluorescent microscope using a 63x objective. Unless indicated, droplets settled on the glass coverslip were imaged. Images were processed and analysed with Fiji Is Just ImageJ (FIJI).

#### Identification of proteins partitioning within BAZ2A condensates

The nuclear extract was prepared as described previously.^25^ Briefly, 5x10^7^ wild-type ESCs were collected by trypsinization, centrifugated at 210xg for 5 minutes and resuspended in 15 ml of cytoplasmic extract buffer (20 mM HEPES, 10 mM KCl, 5 mM MgCl2, 1 mM EDTA, 1 mM DTT, cOmplete™ Protease Inhibitor Cocktail) supplemented with 0.1% of NP40. After 5 minutes of incubation on ice, nuclei were centrifugated at 500xg at 4°C for 5 minutes and washed three times in 10 ml of cytoplasmic extract buffer. Nuclei were further resuspended in 2 ml of nuclear extract buffer (20 mM Tris-HCl, 420 mM NaCl, 1.5 mM MgCl2, 0.2 mM EDTA, 1 mM PMSF, 25% glycerol, 1 mM DTT, cOmplete™ Protease Inhibitor Cocktail) and rotated for 1 hour at 4°C. Nuclear lysates were clarified at 22,000xg at 4°C for 30 minutes. The supernatant was dialyzed overnight at 4°C with dialysis buffer (20 mM Tris-HCl, 75 mM NaCl, 1.5 mM MgCl2, 0.2 mM EDTA, 1 mM PMSF, 10% glycerol, 1 mM DTT) using Slide-A-Lyzer cassette G1 (Thermo, 66380).

Purified recombinant proteins (mGFP-BAZ2A_N_ or BAZ2A_N/ΔTAM_) and the nuclear extract were clarified at 22,000xg at 4°C for 30 minutes to remove any insoluble material. Three independent replicates were performed per condition as described. 30 μg of nuclear extract (150 μl), 20 μg of recombinant protein (40 μl), and 1% of PEG8000 were mixed in a LoBind 1.5 ml Eppendorf tube (022431081), for a total volume of 200 μl. For the negative control, 40 μl of protein dialysis buffer (50 mM Tris-HCl pH 7.5, 500 mM KCl, 5% Glycerol, 1 mM DTT) was added instead of the recombinant protein. The mix was incubated 30 minutes at room temperature and centrifuged 10,000xg at 4°C for 10 minutes. The supernatant was carefully removed, and the pellet was resuspended in 20 μl of 50mM Tris-HCl pH 7.5 containing 10% SDS and transferred to a new 1.5 ml tube. For mass-spectrometry analysis, samples were brought to a final concentration of 4% SDS and boiled for 10 min at 95°C followed by mechanical lysis using a tissue homogenizer (2x2min cycles at 30Hz) and high-intensity focused ultrasound (HIFU). Peptide/Protein concentration was estimated using the Lunatic UV/Vis absorbance spectrometer (Unchained Lab). Proteins were reduced and alkylated by adding Tris(2-carboxyethyl) phosphine and 2-Chloroacetamide to a final concentration of 5 mM and 15 mM, respectively. Samples were incubated for 30 min at 30°C; 700 rpm and light-protected and diluted with pure Ethanol to reach a final concentration of 60% EtOH (v/v). The following steps were carried out on a KingFisher Flex System (Thermo Fisher Scientific). The corresponding amount of carboxylated magnetic beads (hydrophobic and hydrophilic) were added to the samples. After binding the proteins to the beads for 30 minutes at RT, beads were washed 3 times with 80% EtOH. For the enzymatic digestion, beads were added to trypsin in 50 mM TEAB. Samples were digested overnight at 37°C and the remaining peptides were extracted from beads with H_2_O. The two elutions were combined and dried down. The digested samples were dissolved in aqueous 3% Acetonitrile with 0.1% formic acid, and the peptide concentration was estimated with the Lunatic UV/Vis absorbance spectrometer (Unchained Lab). Peptides were separated on a M-class UPLC and analyzed on a Orbitrap mass spectrometer (Thermo). Basic quality check of raw LC-MS data (TIC, BPC, lockmass correction, iRT peptide signals) was performed using rawDiag shiny application.^55^ LC-MS data processing using b-fabric app 295 - FragPipe-RESOURCE. The app executes a Philosopher-based LFQ-MBR workflow using the Philosopher CLI.^56^ Label free quantification was done using the IonQuant software tool.^57^

#### GST-pulldown

The purified GST-tagged proteins (GST-BAZ2A_N_ or GST-mGFP) were thawed on ice and pre-incubated overnight at 4°C with 20 μl Glutathione Sepharose 4B beads in AM100 buffer (20 mM Tris-HCl pH 7.5, 100 mM KCl, 20 mM, 5 mM MgCl_2_, 0.2 mM EDTA) supplemented with cOmplete™ Protease Inhibitor Cocktail. The mGFP-tagged protein (mGFP-BAZ2A_N_) were thawed on ice and pre-cleared overnight at 4°C with 40 μl Protein A Sepharose CL-4B resin (Cytivia, 17078001) in AM200 buffer (20 mM Tris-HCl pH 7.5, 200 mM KCl, 20 mM, 5 mM MgCl_2_, 0.2 mM EDTA) supplemented with with cOmplete™ Protease Inhibitor Cocktail. The beads were spun down and the pre-cleared mGFP-BAZ2A_N_ protein solution was incubated with the GST-tagged proteins bound to the Glutathione Sepharose 4B beads, for 4 hours at 4°C. The beads were washed 3 times for 5 minutes at 4°C with EBC buffer (50 mM Tris-HCl, 300 mM NaCl, 0.5% NP-40, 5 mM DTT, pH 7.5), resuspended in Laemmli loading buffer and incubated at 95°C for 5 minutes. The samples were analysed by SDS-PAGE and Western blotting.

#### Immunoprecipitation

Cells were collected and the nuclei were isolated as previously described.^24^ Cell nuclei lysed for 30 minutes at 37°C in MNase digestion buffer (50 mM Tris–HCl pH 7.5, 0.3 M Sucrose, 30 mM KCl, 7.5 mM NaCl, 4 mM MgCl_2_, 1 mM CaCl_2_, 0.125% NP-40) freshly supplemented with 0.25% NaDeoxycholate, cOmplete™ Protease Inhibitor Cocktail and 2 Unit of MNase (Roche, 10107921001) per million cells. The nuclear extract was then incubated for 10 minutes at 4°C with 200 mM of NaCl to further solubilize chromatin-bound proteins. Samples were then spun down and the cleared supernatant was transferred to a new tube. 20% of the lysate was resuspended in Laemmli loading buffer to use as input while the rest was incubated overnight at 4°C with 30 μl of Anti-HA magnetic beads (Pierce™, 88836) or with 20 μl GFP-Trap® Magnetic Agarose beads (ChromoTek). The beads were washed 3 times at 4°C for 10 to 20 minutes with washing buffer (20 mM HEPES pH 7.6, 20% Glycerol, 200 mM NaCl, 1.5 mM MgCl_2_, 0.2 mM EDTA, 0.02% NP-40), resuspended in Laemmli loading buffer and incubated at 95°C for 5 minutes. The samples were analysed by SDS-PAGE and Western blotting.

#### Size exclusion chromatography and silver staining

For the size exclusion chromatography, the recombinant proteins were freshly purified the day before performing the experiment and concentrated up to 1 mg/ml using a Amicon Ultra-0.5 Centrifugal Filter Unit (Millipore, UFC5050). Protein quality and amount was further checked by SDS-PAGE and Coomassie staining prior to load the protein into a Superose® 6 Increase 3.2/300 column (Cytiva, 29-0915-9). The fractions were collected and prepared for polyacrylamide gel electrophoresis, by adding Laemmli loading buffer (10% glycerol, 10 mM Tris pH 6.8, 0.1 mg/ml bromophenolblue, 2% β-mercaptoethanol, 2% SDS). For the non-denaturing PAGE, no SDS was used in the loading buffer and in the preparation of the polyacrylamide gel and the running buffer. The proteins were detected by silver staining of gels.

#### Negative staining

Continuous carbon coated 300 mesh Cu grids were stained with 2% uranyl acetate (UA). Briefly, the grids were glow discharged for 50 seconds using 20 mA current. Then, 5 μl of the sample solution was applied on the grid and incubated for 60 seconds. The liquid was blotted away and a drop of 2% UA solution placed and blotted immediately. Then, a second drop of UA was deposited, incubated for 60 seconds and blotted away. The grids were imaged in a 120 kV Tecnai Spirit microscope (FEI) using a pixel size of 3.3 Å.

#### *In vitro* synthesis of pRNA and *Malat1*

Topo2.1 plasmids with insertion of pRNA (210 nt) and Control-RNA (197 nt) sequences were previously described.^22^ For *Malat1* cloning, the plasmid encoding the mouse *Malat1* sequence, a kind gift *of* K. V. Prasanth,^31^ was used as template to amplify by PCR the region of interest (197 nt), which was then cloned into a Topo2.1 vector. BamHI-linearized plasmids were *in vitro* transcribed with T7 RNA polymerase (Thermo Fisher, EP0111). Synthesized RNA transcripts were treated with DNase I to remove the linearized plasmid, verified by agarose gel electrophoresis and purified using TRIzol reagent (Life Technologies). The obtained RNA was further verified by agarose gel electrophoresis and stored at -80°C.

#### Competition EMSA

Radiolabeled RNA-Control was synthesized by T7 RNA polymerase using UTP [α-32P]. After treatment with DNase I and RNA purification, 20,000 cpm of RNA-Control were incubated for 15 minutes on ice with 40 ng recombinant BAZ2A_332-723_ in EMSA buffer (20mMTris-HCl [pH 8.0], 5 mM MgCl_2_, 100 mM KCl, and 0.2 mM EDTA). Cold competitor RNA at the indicated amounts was added, and incubation was continued for 30 minutes. RNA-protein complexes were analyzed by electrophoresis on 6% (w/v) native polyacrylamide gels and depicted with autoradiography.

#### Chromatin immunoprecipitation (ChIP)

ChIP analysis was performed as previously described.^21^ Briefly, 1% formaldehyde was added to cultured cells to cross-link proteins to DNA. For H3K27me3 ChIPs, isolated nuclei were then lysed and sonicated using a Bioruptor® Pico ultrasonic cell disruptor (Diagenode) to shear genomic DNA to an average fragment size of 200 bp. 20 μg of chromatin was diluted to a total volume of 500 μl with ChIP buffer (16.7 mM Tris–HCl, pH 8.1, 167 mM NaCl, 1.2 mM EDTA, 0.01% SDS, 1.1% Triton X-100) and pre-cleared with 10 μl packed Sepharose beads for 2 hours at 4°C. Pre-cleared chromatin was incubated overnight with ChIP-grade antibody raised against H3K27me3. The next day, Dynabeads™ Protein A (Invitrogen, 10002D) were added and incubated for 4 hours at 4°C. After washing, bound chromatin was eluted with the elution buffer (1% SDS, 100 mM NaHCO_3_). Upon Proteinase K (Thermo Fisher Scientific) digestion (50°C for 3 hours) and reversion of cross-linking (65°C, overnight), DNA was purified with phenol/chloroform, ethanol precipitated and quantified by qPCR using the primers listed in Table S5.

For BAZ2A ChIPs, crosslinked chromatin has been fragmented into mono-nucleosomes through digestion with MNase. Briefly, isolated and crosslinked nuclei were MNase digested in 400μl MNase digestion buffer (0.3M Sucrose, 50mM Tris pH 7.5, 30mM KCl, 7.5mM NaCl, 4mM MgCl_2_, 1mM CaCl_2_, 0.125% NP-40, 0.25% NaDeoxycholate, supplemented with cOmplete™ Protease Inhibitor Cocktail (Roche)) with 100U MNase (Roche) at 37°C for 30 minutes. The digestion was then stopped with 5mM EDTA and the digested chromatin was solubilized in 1% SDS and three pulses of 30 seconds sonication using a Bioruptor® Pico ultrasonic cell disruptor (Diagenode). 200μg of pre-cleared chromatin was immunopurified with incubation of 20μl of Anti-FLAG® M2 Affinity Gel (Sigma, A2220) over night. The samples were subsequently washed, eluted and the DNA was purified as for histone ChIPs. ChIP-qPCR measurements were performed with KAPA SYBR® FAST (Sigma) on a Rotor-Gene Q (Qiagen) always comparing enrichments over input samples. Primer sequences are listed in Table S5.

For ChIPseq normalization with *D. melanogaster* spike-in chromatin, the samples were prepared and processed as above but with the addition of 200 ng of *D. melanogaster S2* chromatin and 0.5 μg of H2Av antibody (Active Motif, 61686) for BAZ2A ChIP and 50 ng of *D. melanogaster S2* chromatin and 0.2 ug of H2Av antibody for H3K27me3 ChIP.

For ChIPseq analyses, the quantity and quality of the isolated DNA was determined with Qubit® 4 Fluorometer (Life Technologies). The NEBNext® Ultra™ II DNA Library Prep for Illumina (New England Biolabs, E7645S and E7645L) was used following the manufacturer’s protocol. Briefly, ChIP and input samples (10 ng) were end-repaired and polyadenylated before the ligation of Illumina compatible adapters. The adapters contain the index for multiplexing. The quality and quantity of the enriched libraries were validated using Qubit® 4 Fluorometer and 4200 TapeStation System (Agilent). Sequencing was performed on an Illumina NovaSeq6000 machine with single-end 100 bp reads.

#### ChIPseq data analysis

ChIPseq reads were aligned to the mouse mm10 reference genome using Bowtie2 (version 2.5.0; Langmead and Salzberg^58^). Read counts were computed and normalized using “bamCoverage” from deepTools (version 3.5.0; Ramírez et al.^59^) using a bin size of 50bp. “computeMatrix” from deepTools was used to generate all heat maps and plot profiles. BAZ2A bound regions were defined using SICER (version 1.1; Zang et al.^60^) by comparing the FLAG ChIPs of tagged BAZ2A ESCs and input. Spearman’s correlation plots were generated by calculating read coverage for 100kb bin regions using “multiBigwigSummary” from deepTools after removal of blacklist regions. H3K27me3 (GSM3061158), H3K27ac (GSM3061162)^24^ and *Malat1*-DNA interactions dataset^33^ were taken from published ChIPseq data sets of ESCs. Integrative Genome Viewer (IGV, version 2.15.4)^61^ was used to visualize and extract representative ChIPseq tracks.

#### RNAseq and data analysis

Total RNA was purified with TRIzol reagent (Life Technologies). The quality of the isolated RNA was determined with a Qubit® 4 Fluorometer and 4200 Tape Station system. Only those samples with a 260 nm/280 nm ratio between 1.8–2.1 and a 28S/18S ratio within 1.5-2 were further processed. The TruSeq Stranded mRNA (Illumina) was used in the following steps. Briefly, total RNA samples (100-1000 ng) were polyA enriched and then reverse-transcribed into double-stranded cDNA. The cDNA samples were fragmented, end-repaired and adenylated before ligation of TruSeq adapters containing unique dual indices (UDI) for multiplexing. Fragments containing TruSeq adapters on both ends were selectively enriched with PCR. The quality and quantity of the enriched libraries were validated using Qubit® 4 Fluorometer. The product is a smear with an average fragment size of approximately 260 bp. Libraries were normalized to 10 nM in Tris-HCl 10 mM, pH8.5 with 0.1% Tween 20. The HiSeq 4000 (Illumina) was used for cluster generation and sequencing according to standard protocol. Sequencing was paired end at 2x150 bp or single end 100 bp. The quality of the 120 bp single end reads generated by the machine was checked by FastQC, a quality control tool for high throughput sequence data.^62^ The quality of the reads was increased by applying: a) SortMeRNA^63^ (version 2.1) tool to filter out ribosomal RNA; b) Trimmomatic^64^ (version 0.40) software package to trim the sorted (a) reads. The sorted (a), trimmed (b) reads were mapped against the mouse genome (mm10) using the default parameters of the STAR (Spliced Transcripts Alignment to a Reference, version 2.7.0a).^65^ For each gene, exon coverage was calculated using a custom pipeline and then normalized in reads per kilobase per million (RPKM),^66^ the method of quantifying gene expression from RNA sequencing data by normalizing for total read length and the number of sequencing reads. RNAseq data from ESC+2i and ESC+serum were published in^24^ and can be found in GSE112222.

#### BAZ2A iCLIP

Individual-nucleotide resolution UV-crosslinking and immunoprecipitation (iCLIP) analysis for BAZ2A in ESCs was performed according to Huppertz et al.^67^ with the following modifications. About 2.5x10^6^ ESCs were subjected to 400 mJ/cm^2^ UV-C (254 nm) crosslinking energy. Cells were subsequently scraped on ice and pelleted at 500 g. To control for unspecific RNA binding 2.5x10^6^ ESCs were also subjected to iCLIP analysis without being subjected to UV-crosslinking. The entire procedure was performed in triplicates for each condition. To isolate nuclei cells were resuspended in hypotonic buffer (10 mM HEPES pH 7.6, 1.5 mM MgC2, 10 mM KCl), incubated 10 minutes on ice, then TritonX-100 was added to the final concentration of 0.1%. Upon additional 10 minutes of incubation on ice, nuclei were pelleted by 5 minutes centrifugation at 1600 g at 4°C. Nuclei were lysed into lysis buffer (50 mM Tris-HCl pH 7.4, 100 mM NaCl, 0.1% SDS, 1% NP-40, 0.5% NaDeoxycholate) and treated with TURBO™ DNase and RNase I (ThermoFisher, AM2238 and EN0601) (high RNase condition 1:50; low RNase condition 1:400) for 3 minutes at 37°C at 1100 rpm. To properly solubilize BAZ2A, lysate was subjected to high salt extraction by adding NaCl to final concentration of 400 mM. Upon 10 minutes incubation on ice, lysate was then centrifuged 10 minutes at 21000 g at 4°C. Salt concentration was diluted back to physiological 150 mM NaCl by adding iCLIP lysis buffer lacking NaCl.

Immunoprecipitation was performed by incubating lysate with the Anti-FLAG® M2 Affinity Gel agarose beads overnight at 4°C under rotation. Beads were washed subsequently twice in High-salt wash (50 mM Tris-HCl pH 7.4, 1 M NaCl, 1 mM EDTA, 1% NP-40, 0.1% SDS, 0.5% NaDeoxycholate), twice in 0.8 M Urea wash (0.8 M Urea, 20 mM Tris pH 7.4, 250 mM NaCl, 1 mM EDTA, 1% NP-40, 0.1% SDS, 0.5% NaDeoxycholate), and twice in PNK buffer (20 mM Tris pH 7.4, 10 mM MgCl_2_, 0.2% Tween-20). All washes were performed by 5 minutes incubation at 4°C under rotation followed by 3 min centrifugation at 1300 g at 4°C. RNA 3’ end dephosphorylation and L3 adapter ligation steps were performed on beads according to original protocol. 90% of the beads were subjected to RNA 5’ end labeling in a total volume of 20 μl of hot PNK mix 2 μl 10× PNK buffer, 16.5 μl H_2_O, 1 μl PNK, 0.5 μl of 10μCi/μl γ-^32^P-ATP). Upon 10 min incubation at 37°C at 1100 rpm, beads were quickly washed in PNK buffer.

Immunoprecipitated BAZ2A-RNA complexes were eluted from beads by adding a mix containing 20 μl PNK buffer, 6 μl of 4× LDS NuPAGE sample buffer and 1 μl of 1 M DTT and incubated 10 minutes at 70°C at 1100 rpm. Eluted material was then subjected to SDS-PAGE on a 3-8% TrisAcetate pre-casted gel for 1 hour at 150 V followed by semidry transfer on nitrocellulose membrane using the iBlot™ Gel Transfer Device (Invitrogen). Membrane was then exposed for 1hour or overnight to a Fuji film for autoradiography. Isolation of RNA species associated with BAZ2A from nitrocellulose membrane and reverse transcription were performed according to original protocol. Crosslinked samples were reverse transcribed using the RT#Clip primer #1 (5Phos/NNAACCNNNAGATCGGAAGAGCGTCGTGgatcCTGAACCGC), whereas the RT#Clip primer #3 (5Phos/NNATTGNNNAGATCGGAAGAGCGTCGTGgatcCTGAACCGC) was used for non-crosslinked samples. Upon alkaline hydrolysis, precipitated cDNA was subjected to gel purification on a 6% TBE-UREA pre-casted gel according to original protocol. Bands of 80-120 nt were cut out and the gel pieces were then placed into a 0.5 ml microtube, where a hole on its bottom was previously made using a 21 g needle. Microtube was placed on a 2 ml tube and subjected to centrifugation for 2 minutes at max speed at room temperature. Crushed gel pieces were then supplemented with 400 μl TE buffer and incubated 30 minutes at 50°C at 1100 rpm. Eluted material was then passed into a Costar® Spin-X® Centrifuge Tube Filters (Corning, 8161) with two glass pre-filters by spinning 1 minute at max speed at room temperature, and cDNA was subsequently isolated by phenol/chloroform extraction following original protocol’s instructions. Ligation of the RT#Clip primer to the 5’ end of the cDNA via circularization and re-linearization steps were performed according to the original protocol. PCR optimization was performed as indicated and 28 cycles amplification was selected for preparative PCR. Since quality check on gel clearly showed no amplification products for non-crosslinking samples, each crosslinked replicate was pulled in a 1/1 volume ratio with its corresponding non-crosslinked replicate. Libraries were then size-selected and further purified using the BluePippin system (Sage Science) according to manufacturer’s instructions. Upon quantification and quality check on 4200 TapeStation System (Agilent), samples were subjected to next generation sequencing.

For BAZ2A-RNA immunoprecipitation (RIP), 7x10^6^ cells were crosslinked and processed as described above. BAZ2A-RNA complexes were immunoprecipitated by incubating the nuclear lysate with the Anti-FLAG® M2 Affinity Gel (Sigma-Aldrich) overnight at 4°C under rotation. 4% of the lysate was kept as input and stored at -80°C. Beads then were washed twice for 5 minutes at 4°C with High-salt wash, Urea wash and PNK buffers. Each wash was followed by 3 minutes centrifugation at 1300 g at 4°C. The RNA was eluted by incubating the beads in PK buffer (100 mM Tris pH 7.4, 50 mM NaCl, 10 mM EDTA) supplemented with 0.2 mg of Proteinase K and incubated 20 minutes at 37°C shaking at 1100 rpm. Same volume of PK buffer with 7 M Urea was added and incubated for another 20 minutes at 37°C shaking at 1100 rpm. RNA from both input and RIP samples was subsequently isolated by phenol/chloroform extraction following original protocol’s instruction. RNA samples were then reverse transcribed into double-stranded cDNA which was quantified by qPCR comparing enrichments over input samples. Primer sequences are listed in Table S5.

#### iCLIP data analysis

iCLIP data was generated for crosslinked and noncrosslinked background control samples in triplicates (17 - 29x10^6^ reads). iCLIP reads were demultiplexed and trimmed of adaptors using Flexbar (version 3.5). Reads were then mapped to the mouse genome (mm10) using STAR (version 2.6). Mapped reads were deduplicated using UMI-tools (version 0.5.1). Peaks were called using omniCLIP (version 0.1.0)^68^ with default parameters. Mouse gene annotations, including 5’ UTR, CDS, 3’ UTR, splice sites, introns were downloaded from Ensembl (version 100). Enrichment of gene annotations in BAZ2A-iCLIP peaks was tested using Genome Association Tester (GAT, version 1.3.6). Enriched sequence motifs within BAZ2A-iCLIP peaks were discovered using DREME, selecting only for motifs between 7-10 nucleotides in length, on the sense direction of the RNA transcript, and with e-value<0.05. TOMTOM was used to match BAZ2A motifs to known mouse RNA motifs. Enriched sequence motifs within BAZ2A iCLIP sites were discovered using DREME, selecting only for motifs between 7-10 nucleotides in length, on the sense direction of the RNA transcript, and with e-value<0.05. TOMTOM was used to match BAZ2A motifs to known mouse RNA motifs.

#### SRRM2 TSA-seq and data analysis

SRRM2 TSA-seq analysis was performed according to Chen et al.^34^ with the following modifications. In short, 1% formaldehyde was added to 7.5x10^6^ mouse ESCs to cross-link proteins to DNA. Cells were then permeabilized in PBS with TritonX-100 0.5% for 5 minutes on ice and 10 minutes at room temperature. Next, cells were resuspended in 1.5% H_2_O_2_ to quench the endogenous peroxidases by slowly nutating at room temperature for 1 hour. After three washes with PBS-TritonX-100 0.1% (PBST), cells were resuspended in GS blocking buffer (1% BSA in PBST) for 1 hour at room temperature. Cells were then incubated with antibody anti-SRRM2 (Novus Biologicals, NBP2-55697) 1:500 in GS blocking buffer overnight at 4°C. For the no-antibody control sample, cells were incubated in GS blocking buffer only. After washing, cells were incubated with HRP conjugated secondary antibody (Vector Laboratories, PI-1000-1) 1:1000 in GS blocking buffer for 2 hours at room temperature. Cells were then incubated in Buffer A (50% sucrose, 0.0015% H_2_O_2_, 1:2500 tyramide-biotin 8 mM (Sigma-Aldrich, SML2135) for 10 minutes nutating at room temperature.

For monitoring TSA labeling by immunostaining, a small aliquot of each sample was placed on a glass coverslip, air dried for few minutes and fixed on the coverslip with 3.7% formaldehyde in PBS for 10 minutes at room temperature. After washing in PBST three times, coverslips were incubated 30 minutes at room temperature with streptavidin-Alex Fluor 594 (Invitrogen, S11227) 1:1000 and Alex Fluor 488 secondary antibody (Thermo Fisher, A11034) 1:1000 in GS blocking buffer. After washing, the coverslips were mounted using VECTASHIELD® Antifade Mounting Medium with DAPI.

To shear the DNA, cells were resuspended in MNase digestion buffer (0.3M Sucrose, 50mM Tris pH 7.5, 30mM KCl, 7.5mM NaCl, 4mM MgCl_2_, 1mM CaCl_2_, 0.125% NP-40, 0.25% NaDeoxycholate, supplemented with cOmplete™ Protease Inhibitor Cocktail (Roche)) with 100U MNase (Roche) at 37°C for 30 minutes. The digestion was then stopped with 5mM EDTA and the digested chromatin was solubilized in 200mM NaCl and five pulses of 30sec sonication using a Bioruptor® Pico ultrasonic cell disruptor (Diagenode). For the affinity purification of biotinylated DNA, 10μl/10^6^ cells of Dynabeads™ M-270 streptadivin (Invitrogen) were washed in Wash and Binding (W&B) buffer (5 mM Tris pH 7.5, 0.5 mM EDTA, 1 M NaCl) before being added to the samples. Beads were then washed four times in W&B buffer, TSE 500 (20 mM Tris pH 8, 1% TritonX-100, 0.1% SDS, 2 mM EDTA pH 8, 500 mM NaCl), again in W&B buffer and then in TE buffer. After the final wash, beads were resuspended in Proteinase K buffer (10 mM Tris pH 7,5, 10 mM EDTA pH 8, 0.5% SDS) supplemented with free biotin 0.1 mM and 40 μg of Proteinase K (Thermo Scientific) and incubated at 50°C for 3 hours and reverse cross-linked at 65°C, overnight. DNA was isolated by phenol/chloroform extraction following original protocol’s instructions. Quantity and quality of the isolated DNA was determined with Qubit® 4 Fluorometer (Life Technologies). The NEBNext® Ultra™ II DNA Library Prep for Illumina (New England Biolabs, E7645S and E7645L) was used following the manufacturer’s protocol. Briefly, TSA and input samples (10 ng) were end-repaired and polyadenylated before the ligation of Illumina compatible adapters. The adapters contain the index for multiplexing. The quality and quantity of the enriched libraries were validated using Qubit® 4 Fluorometer and 4200 TapeStation System (Agilent). Sequencing was performed on an Illumina NovaSeq6000 machine with single-end 100 bp reads.

TSA-seq reads were aligned to the mouse mm10 reference genome using Bowtie2 (version 2.5.0; Langmead and Salzberg^58^). Read counts were computed and normalized using “bamCoverage” from deepTools (version 3.5.0; Ramírez et al.^59^) using a bin size of 50bp. “bamCompare” from deepTools was used to compute the log_2_ ratio for each bin after normalized the data for sequencing depth. Integrative Genome Viewer (IGV, version 2.15.4)^61^ was used to visualize and extract representative TSA-seq tracks.

### Quantification and statistical analysis

Statistical details of biological triplicate experiments can be found in the corresponding figure legends. Data were represented as box plots, which show the minimum and maximum values. The horizontal line within the boxes represents the mean value, and error bars represent ±SD. Data represented as bardiagram show the mean and the +SD.

## Supplemental information

Document S1. Figures S1–S7

Table S1. Mass-spec analysis of BAZ2A droplets, related to Figure 2

Table S2. RNA-seq of ESC + 2i expressing BAZ2A_WT_ and BAZ2A_ΔTAM_, related to Figure 4

Table S3. GO analysis of genes up- or downregulated in RNA-seq of ESC + 2i expressing BAZ2A_ΔTAM_ relative to ESCs expressing BAZ2A_WT_, related to Figure 4

Table S4. List of genes containing BAZ2A-iCLIP signals, related to Figure 5

Table S5. List of primers, antibodies, and datasets used in this study

Document S2. Article plus supplemental information
