# Supplementary material for: An RNA-dependent and phase-separated active subnuclear compartment safeguards repressive chromatin domains
Source: Mol Cell. 2024 May 2;84(9):1667–1683.e10. doi: 10.1016/j.molcel.2024.03.015 (PMC11065421; doi:10.1016/j.molcel.2024.03.015)
Supplement: Document S1. Figures S1–S7 [file mmc1.pdf]

Molecular Cell, Volume *84*

Supplemental information

An RNA-dependent and phase-separated active subnuclear compartment safeguards repressive chromatin domains

Luigi Lerra, Martina Panatta, Dominik Bär, Isabella Zanini, Jennifer Yihong Tan, Agnese Pisano, Chiara Mungo, Célia Baroux, Vikram Govind Panse, Ana C. Marques, and Raffaella Santoro

Supplementary Figures

An RNA-dependent and phase-separated active subnuclear compartment safeguards repressive chromatin domains

Luigi Lerra[12#] Martina Panatta[12#] Dominik Bär[1] Isabella Zanini[1] Jennifer Yihong Tan[3] Agnese Pisano[4] Chiara Mungo[15] Celia Baroux[6] Vikram Govind Panse[4] Ana C. Marques[3] Raffaella Santoro[1*]

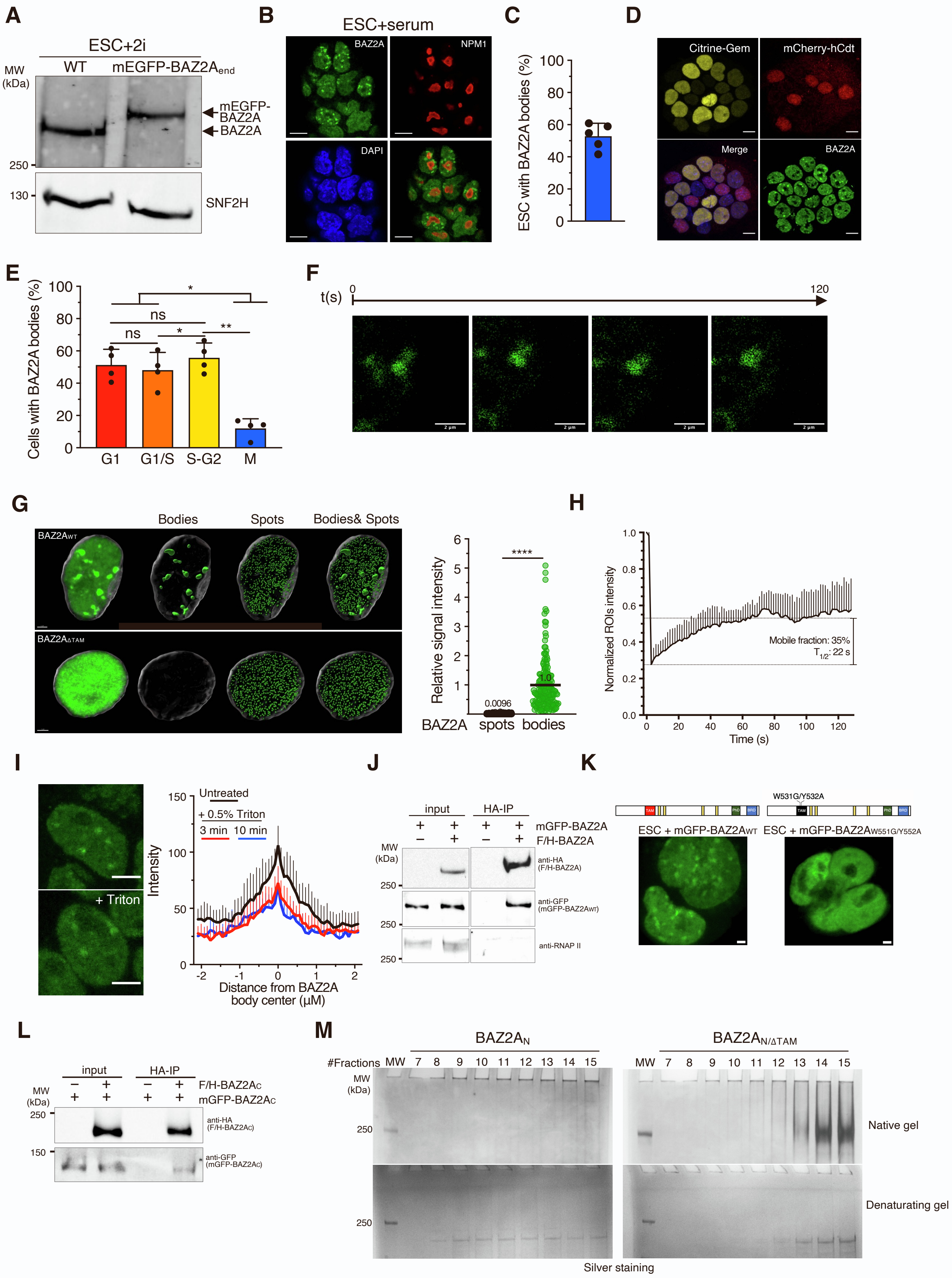
A
ESC+2i
WT
mEGFP-BAZ2Aend
MW (kDa)
mEGFP-BAZ2A
BAZ2A
250
130
SNF2H
B
ESC+serum
BAZ2A
NPM1
DAPI
C
ESC with BAZ2A bodies (%)
100
80
60
40
20
0
D
Citrine-Gem
mCherry-hCdt
Merge
BAZ2A
E
Cells with BAZ2A bodies (%)
*
ns
ns
*
**
G1
G1/S
S-G2
M
F
t(s)
0
120
2 µm
G
Bodies
Spots
Bodies& Spots
BAZ2AWT
BAZ2AΔTAM
Relative signal intensity
****
0.0096
1.0
BAZ2A
spots
bodies
H
Normalized ROIs intensity
Mobile fraction: 35%
T1/2: 22 s
Time (s)
I
+ Triton
Intensity
Untreated
+ 0.5% Triton
3 min
10 min
Distance from BAZ2A body center (µM)
J
input
HA-IP
mGFP-BAZ2A
F/H-BAZ2A
MW (kDa)
anti-HA (F/H-BAZ2A)
anti-GFP (mGFP-BAZ2AWT)
anti-RNAP II
K
W531G/Y532A
ESC + mGFP-BAZ2AWT
ESC + mGFP-BAZ2AW551G/Y552A
L
input
HA-IP
F/H-BAZ2AC
mGFP-BAZ2AC
MW (kDa)
anti-HA (F/H-BAZ2AC)
anti-GFP (mGFP-BAZ2AC)
M
BAZ2AN
BAZ2AN/ΔTAM
#Fractions
MW
7 8 9 10 11 12 13 14 15
Native gel
Denaturating gel
Silver staining

Figure S1 (Related to Figure 1)
Characterization of BAZ2A bodies in ESCs

A. Western blot analysis showing the expression levels of the endogenous BAZ2A and the endogenous BAZ2A tagged with mGFP (mEGFP-BAZ2A$_{end}$). BAZ2A signal was detected with BAZ2A-antibodies. SNF2H serves as loading control.

B. Representative immunofluorescence images showing BAZ2A localization in ESC+serum using antibodies against BAZ2A and the nucleolar marker NPM1. Scale bar is 10 µm.

C. Proportion of ESC+2i with detectable BAZ2A bodies. Data are from five independent experiments. Each experiment consists of the quantification of ESCs containing BAZ2A bodies from 10 ESC colonies.

D. Representative immunofluorescence image of ESC+2i FUCCI stained for BAZ2A. mCherry-human chromatin licensing and DNA replication factor 1 (hCdt) and Citrine-Geminin (Gem) expression correspond to G1 and S-G2 phases, respectively. Scale bar is 5 µm.

E. Average percentage of ESC+2i FUCCI with detectable BAZ2A bodies for the indicated cell cycle phases. Measurements are from four independent experiments. Each experiment consists of at least 20 ESC colonies. Error bars represent s.d. Statistical significance (*P*-values) for the experiments was calculated using paired t-test (* > 0.05; ** > 0.01; ns: non-significant).

F. Snapshots of a live cell imaging time course (120 seconds) of BAZ2A bodies in ESC+mGFP-BAZ2A$_{end}$. Scale bar is 2 µm.

G. 3D-image reconstruction by Imaris showing BAZ2A bodies and BAZ2A spots of 300 nm diameter in ESC+GFP-BAZ2A$_{WT}$ and ESC+GFP-BAZ2A$_{\Delta TAM}$. On the right it is shown the relative signal intensity of BAZ2A in bodies and spots. Statistical significance (*P*-values) was calculated using unpaired t-test (**** > 0.0001).

H. In the right panel the quantification of mobile fractions of mGFP-BAZ2A$_{end}$ from FRAP experiment. Representative live cell images of ESC+mGFP-BAZ2A$_{end}$ relative to the FRAP quantification are shown on the left panel. The mean recovery and standard deviation of 13 bleached regions of interest is shown. Scale bar is 2 µm.

I. Representative images of one ESC+mGFP-BAZ2A$_{end}$ without (upper left panel) or with (lower left panel) 0.05% Triton treatment (3 minutes). On the right panel, the average fluorescence intensity and standard deviation of 15 BAZ2A bodies is shown.

J. Anti-HA immunoprecipitation from HEK293T cells transfected with plasmids expressing F/H-BAZ2A and mGFP-BAZ2A.

K. Representative live-cell image of ESCs transfected with plasmid expressing mGFP-BAZ2A$_{WT}$ and GFP-BAZ2A$_{W551G/Y552A}$. Scale bar is 2 µm.

L. Anti-HA immunoprecipitation from HEK293T cells transfected with plasmids expressing F/H-BAZ2A and mGFP-BAZ2A$_{C}$.

M. Native (upper panels) and denaturing (lower panels) protein gel electrophoreses of SEC fractions (**Figure 1J**) of recombinant BAZ2A$_{N}$ (left) and BAZ2A$_{N/\Delta TAM}$ (right). Proteins were detected by silver staining.

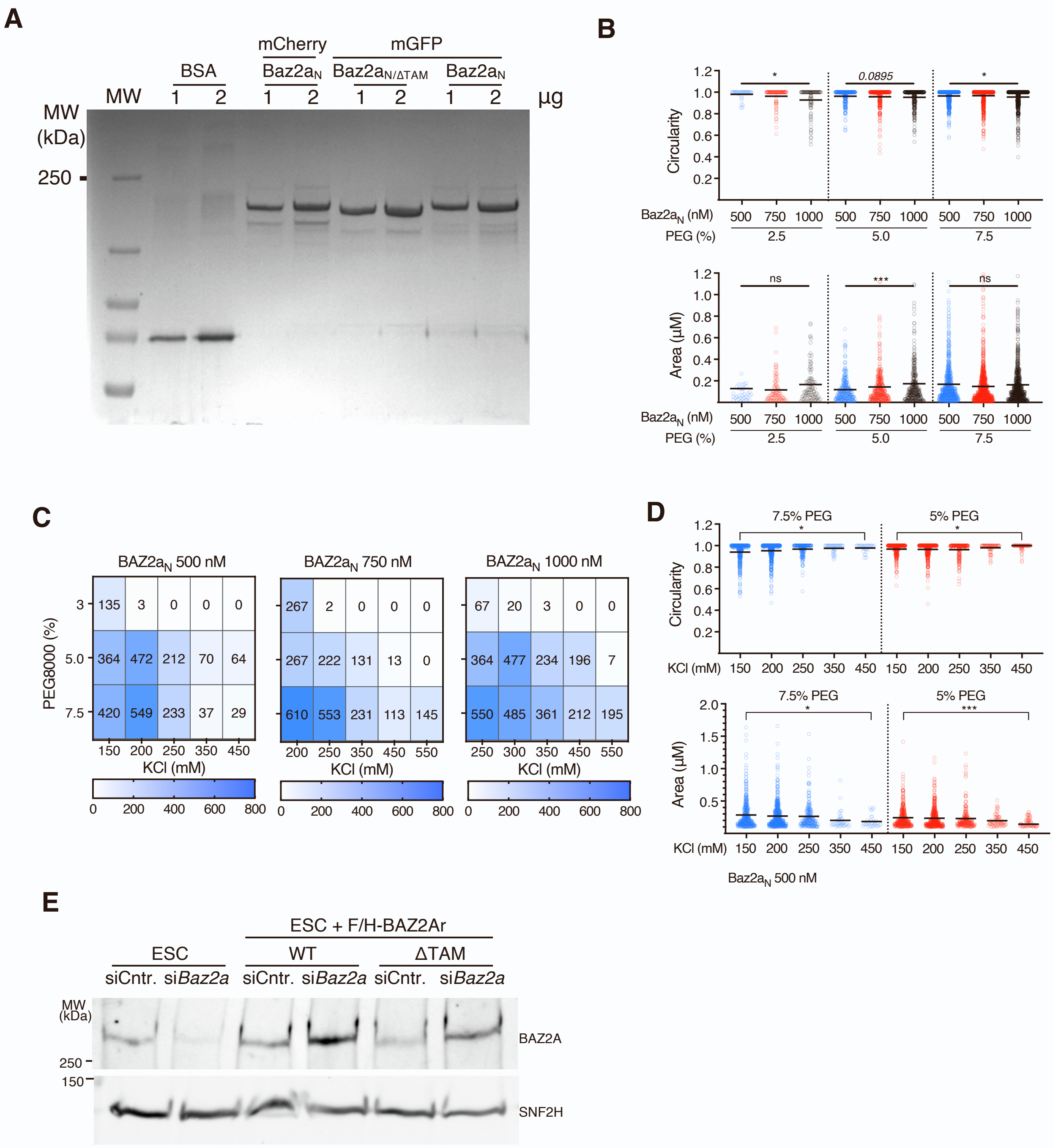

Figure S2 (Related to Figure 2)
Characterisation of BAZ2A droplets

A. Protein gel electrophoresis followed by Coomassie staining of the indicated purified recombinant proteins used for the *in vitro* droplet assays. Albumin (BSA) was used as a loading control to estimate protein concentration.

B. Quantification of circularity and area of BAZ2A droplets formed at increasing protein and PEG8000 concentration. Statistical significance (*P*-values) for the experiments was calculated using Mann-Whitney test (*< 0.05; ***< 0.001).

C. Quantification of number, circularity, and area of BAZ2A droplets formed at increasing BAZ2A, PEG8000 and salt concentration.

D. Quantification of circularity and area of BAZ2A droplets formed using 500 nM Baz2A$_N$ and increasing concentration of PEG and PEG8000 concentration. Statistical significance (*P*-values) for the experiments was calculated using Mann-Whitney test (*< 0.05; ***< 0.001).

E. Western blot showing the expression levels of BAZ2A in the indicated ESC lines upon treatment with siRNA-Control (siCntr.) or siRNA-*Baz2a* (si*Baz2a*).

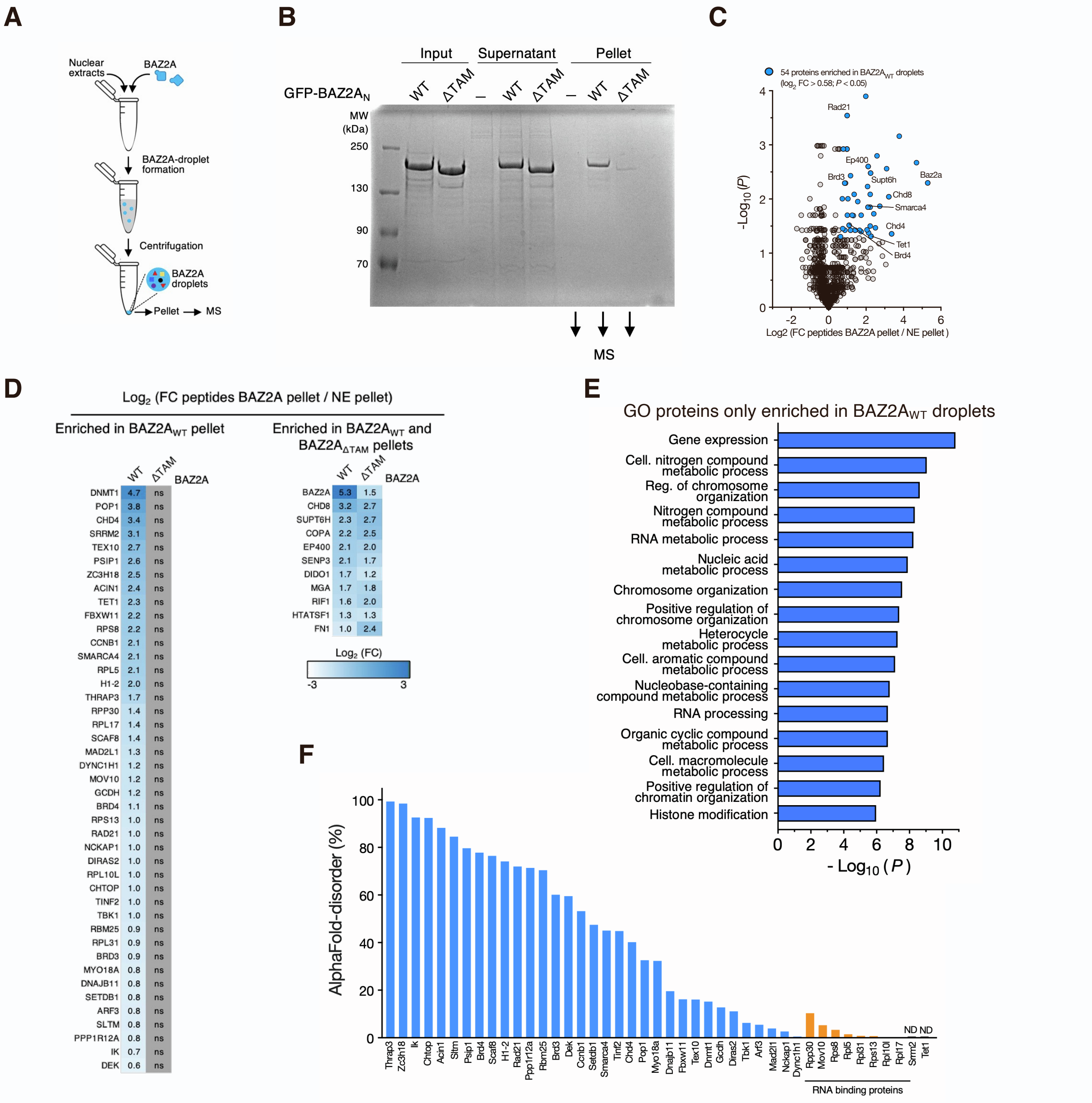

Figure S3 (Related to Figure 2)
Proteins partioning with BAZ2A bodies

A. Illustration of the experimental strategy for the identification of proteins partitioning in to BAZ2A$_{WT}$ bodies.

B. Protein gel stained with Coomassie showing supernatant and pellets of the indicated samples.

C. Volcano plot showing proteins significantly enriched into BAZ2A-droplets relative to pellets of nuclear extracts (NE).

D. Heatmaps showing log$_2$ fold changes (FC) of significantly proteins enriched in BAZ2A$_{WT}$ droplets mixed to nuclear extracts relative to protein found in pellets of BAZ2A$_{ΔTAM}$. Average values of three independent experiments. Statistical significance (*P*-values) was calculated using unpaired two-tailed t-test. ns: non-significant).

E. Gene ontology terms of proteins specifically associated with BAZ2A$_{WT}$ droplets.

F. AlphaFold-disordered values predicting IDR content in proteins specifically associated with BAZ2A$_{WT}$ droplets. Data were generated using MOBIDB [73]. RNA binding proteins, which contained low IDR content, are shown.

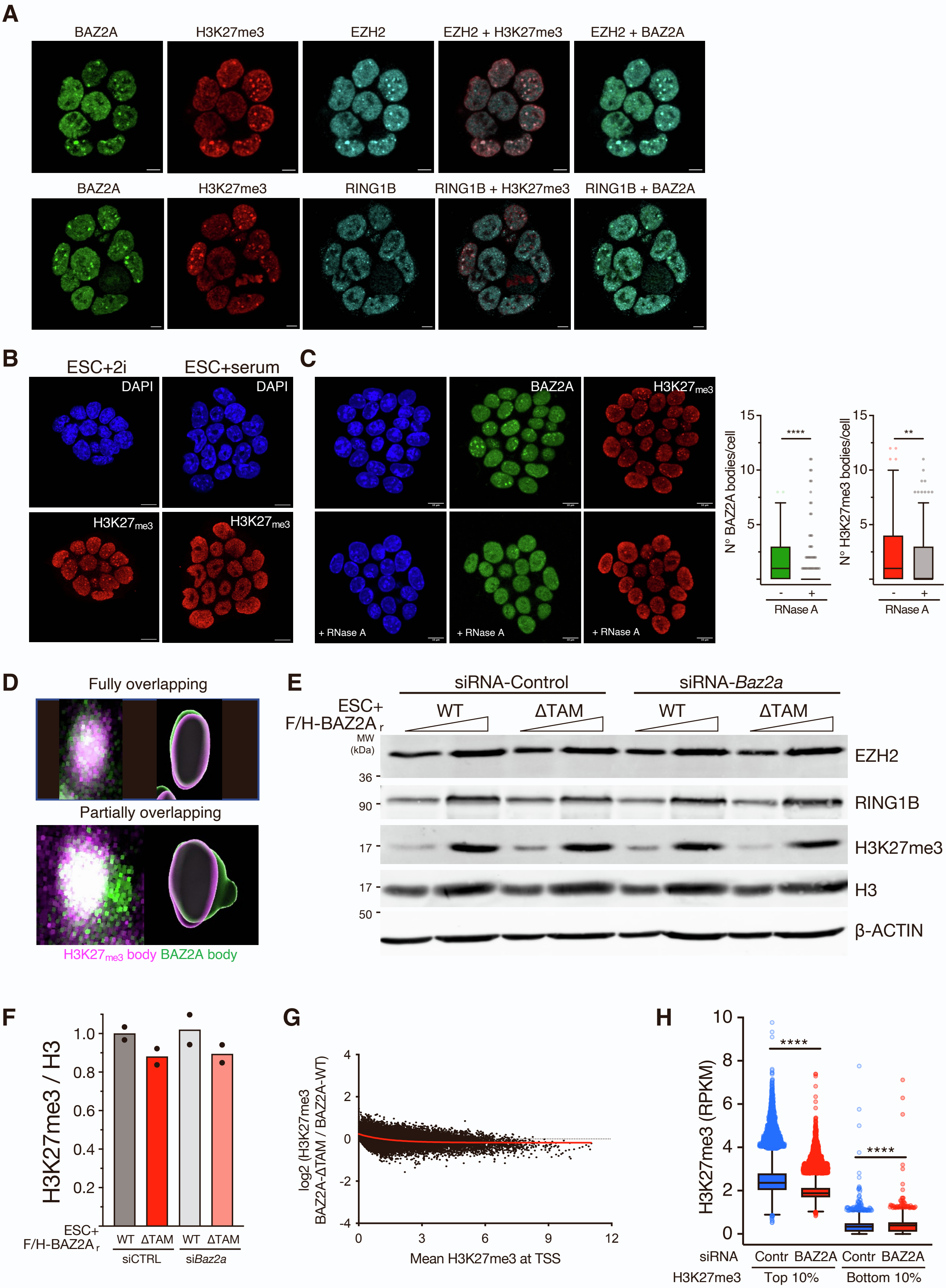
A
BAZ2A
H3K27me3
EZH2
EZH2 + H3K27me3
EZH2 + BAZ2A
BAZ2A
H3K27me3
RING1B
RING1B + H3K27me3
RING1B + BAZ2A
B
ESC+2i
ESC+serum
DAPI
DAPI
H3K27me3
H3K27me3
C
BAZ2A
H3K27me3
+ RNase A
+ RNase A
+ RNase A
N° BAZ2A bodies/cell
N° H3K27me3 bodies/cell
****
**
RNase A
RNase A
D
Fully overlapping
Partially overlapping
H3K27me3 body BAZ2A body
E
siRNA-Control
siRNA-Baz2a
ESC+
F/H-BAZ2Ar
WT
ΔTAM
WT
ΔTAM
MW (kDa)
36
90
17
17
50
EZH2
RING1B
H3K27me3
H3
β-ACTIN
F
H3K27me3 / H3
ESC+
F/H-BAZ2Ar
WT ΔTAM WT ΔTAM
siCTRL
siBaz2a
G
log2 (H3K27me3 BAZ2A-ΔTAM / BAZ2A-WT)
Mean H3K27me3 at TSS
H
H3K27me3 (RPKM)
****
****
siRNA
Contr BAZ2A Contr BAZ2A
H3K27me3
Top 10%
Bottom 10%

Figure S4 (Related to Figure 3)

BAZ2A bodies regulate H3K27me3 chromatin domains

A. Representative immunofluorescence images of BAZ2A, H3K27me3, EZH2, and RING1B in ESC+2i. Scale bar is 8 µm.

B. Representative immunofluorescence images of H3K27me3 in ESC+2i and ESC+serum.

C. Immunofluorescence of mGFP-BAZ2Aend and H3K27me3 treated with RNase A in ESC+2i. On the right, box plots showing the number of BAZ2A and H3K27me3 bodies/cell. Error bars represent s.d. Statistical significance (P-values) for the experiments was calculated using Mann-Whitney test (** < 0.01; **** < 0.0001).

D. Representative 3D reconstruction of BAZ2A and H3K27me3 bodies.

E. Western blot showing EZH2, RING1b, H3K27me3, histone H3, and β-actin protein levels in ESC+F/H-BAZ2ArWT and ESC+F/H-BAZ2ArΔTAM treated with siRNA-Control or siRNA-Baz2a. Each sample was loaded at 1- and 3-fold amounts.

F. Quantifications of H3K27me3 signal normalized to histone H3 levels shown in the western blot of Fig. S4e.

G. Scatter plot showing the changes in H3K27me3 upon BAZ2AΔTAM expression compared to ESC+BAZ2AWT. Mean of normalized H3K27me3 read counts over all TSSs (±1 kb) were calculated. Log2-fold changes of H3K27me3 in ESC+BAZ2AΔTAM vs. ESC+BAZ2AWT are plotted relative to the mean of normalized H3K27me3 occupancies. The red line represents the mean of H3K27me3 fold changes.

H. Levels of H3K27me3 at the 10% top or bottom H3K27me3 regions in ESC+siRNA-Control and ESC+siRNA-BAZ2A from 23. Values are shown as average RPKM of a 10kb bin size region. Error bars represent s.d. Statistical significance (P-values) was calculated using the unpaired two-tailed t test (****<0.0001). Box plots depict the minimum and maximum values. The horizontal line within the boxes represents the mean value.

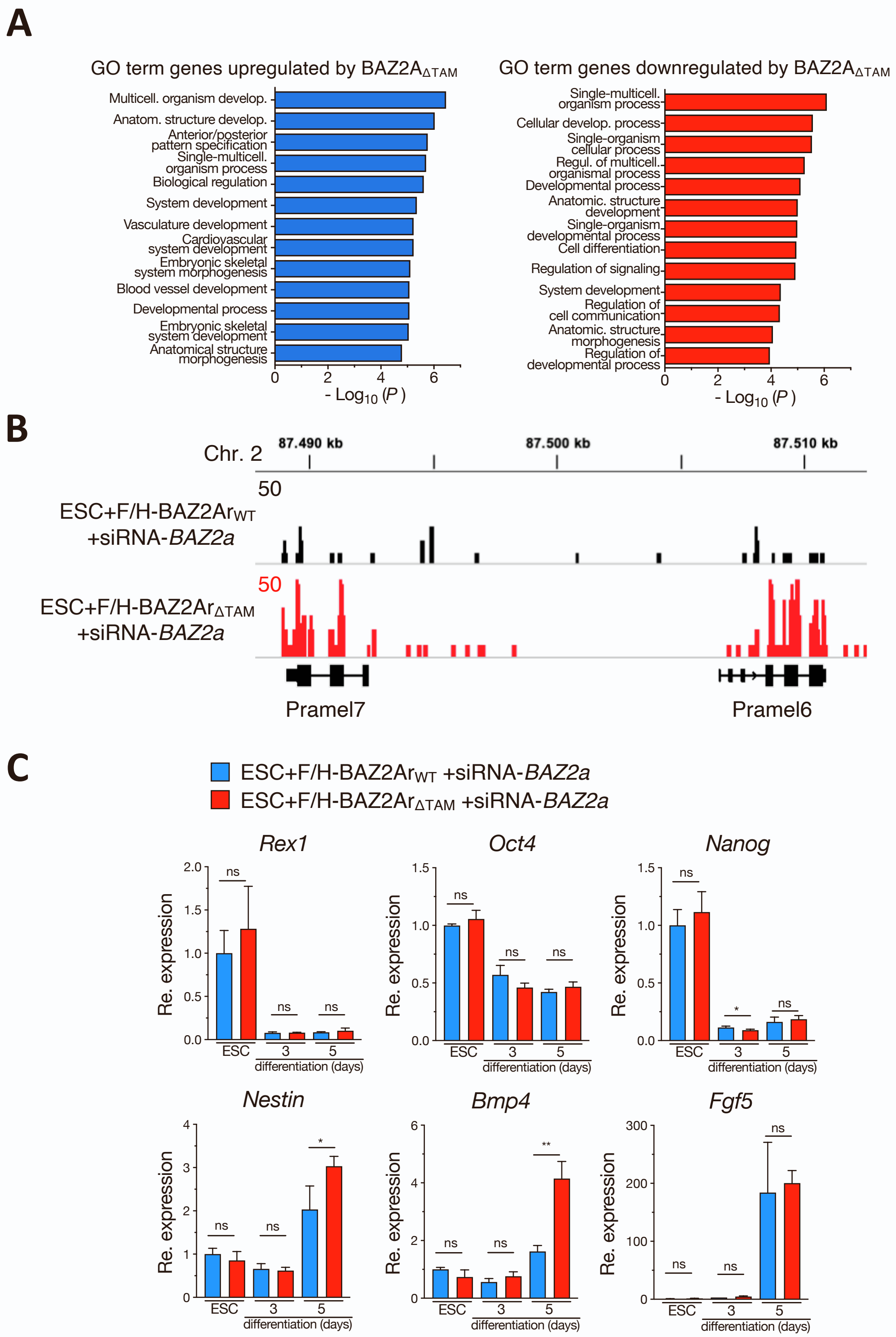

Figure S5 (Related to Figure 4)

BAZ2A$_{\Delta TAM}$ improves the expression of differentiation genes upon ESC differentiation

A. Top 12 gene ontology (GO) terms as determined using DAVID for genes upregulated and downregulated by BAZ2A$_{\Delta TAM}$.

B. Tracks displaying the expression profiles of ground-state *Pramel6* and *Pramel7* genes in ESC+BAZ2AWT and ESC+ BAZ2A$_{\Delta TAM}$.

C. Gene expression analysis of ESC+BAZ2A$_{WT}$ and BAZ2A$_{\Delta TAM}$ showig expression of pluripotency genes Rex1, Nanog, and Oct4) and differentiation genes (*Nestin*: Neuroectoderm; *Bmp4*: Mesoderm; *Fgf5*: Endoderm). mRNA levels were measured by qRT–PCR and normalized to *Hprt* mRNA. Average values of three independent experiments. Error bars represent s.d., and statistical significance (P-values) was calculated using the unpaired two-tailed t-test (* < 0.05; ** < 0.01; ns: non-significant).

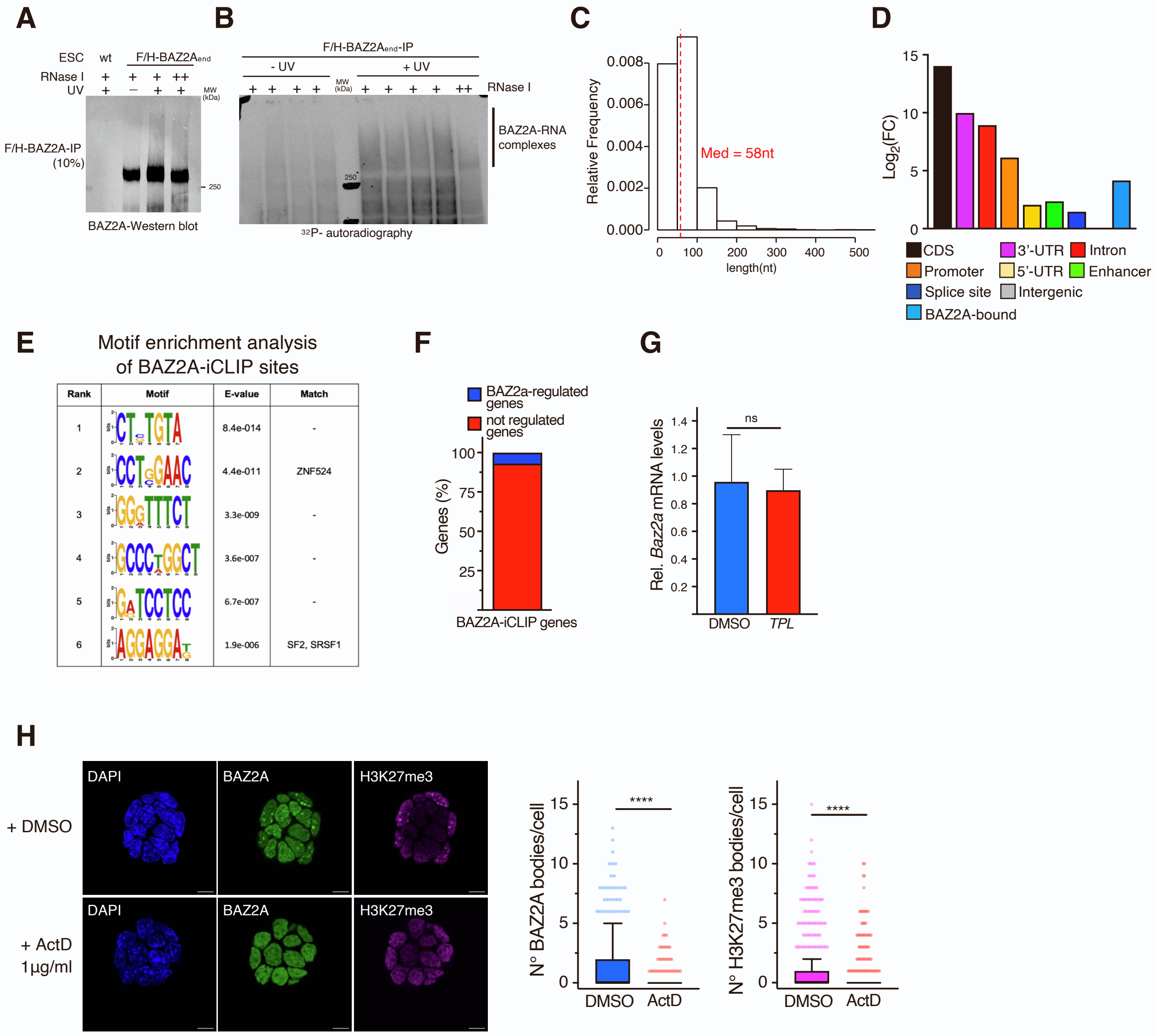

Figure S6 (Related to Figure 5)
BAZ2A associates with RNA

A. HA-immunoprecipitation of ESC+F/H-BAZ2A$_{end}$ with different conditions of UV crosslinking and RNAse I treatment as indicated.

B. Autoradiography of BAZ2A-RNA complexes with ^{32}P-RNA labeled with or without UV crosslinking. High RNAse I treatment of the samples was used as control.

C. Median length of BAZ2A-iCLIP peaks

D. Genome Association Tester (GAT) analysis showing enrichment of BAZ2A-iCLIP sites on the genome.

E. Motif enrichment analysis of BAZ2A-iCLIP sites.

F. Bar diagram showing the proportion of BAZ2A-regulated genes among all genes detected with BAZ2A-iCLIP sites.

G. qRT-PCR showing mRNA levels of *Baz2a* in ESC+2i treated for 4 hours with triptolide. Data are from three independent experiments. Error bars represent s.d., and statistical significance (P-values) was calculated using the paired two-tailed t-test (ns: non-significant)

H. Representative immunofluorescence images showing BAZ2A and H3K27me3 in ESC treated with 1 µg/ml Actinomycin D (ActD) for 4 hours. Scale bar represents 10 µm. Quantifications of the number of BAZ2A- and H3K27me3-bodies/cell are shown in the box plot. Statistical significance (P-values) was calculated using Mann-Whitney test (****<0.0001). Box plots depict the minimum and maximum values.

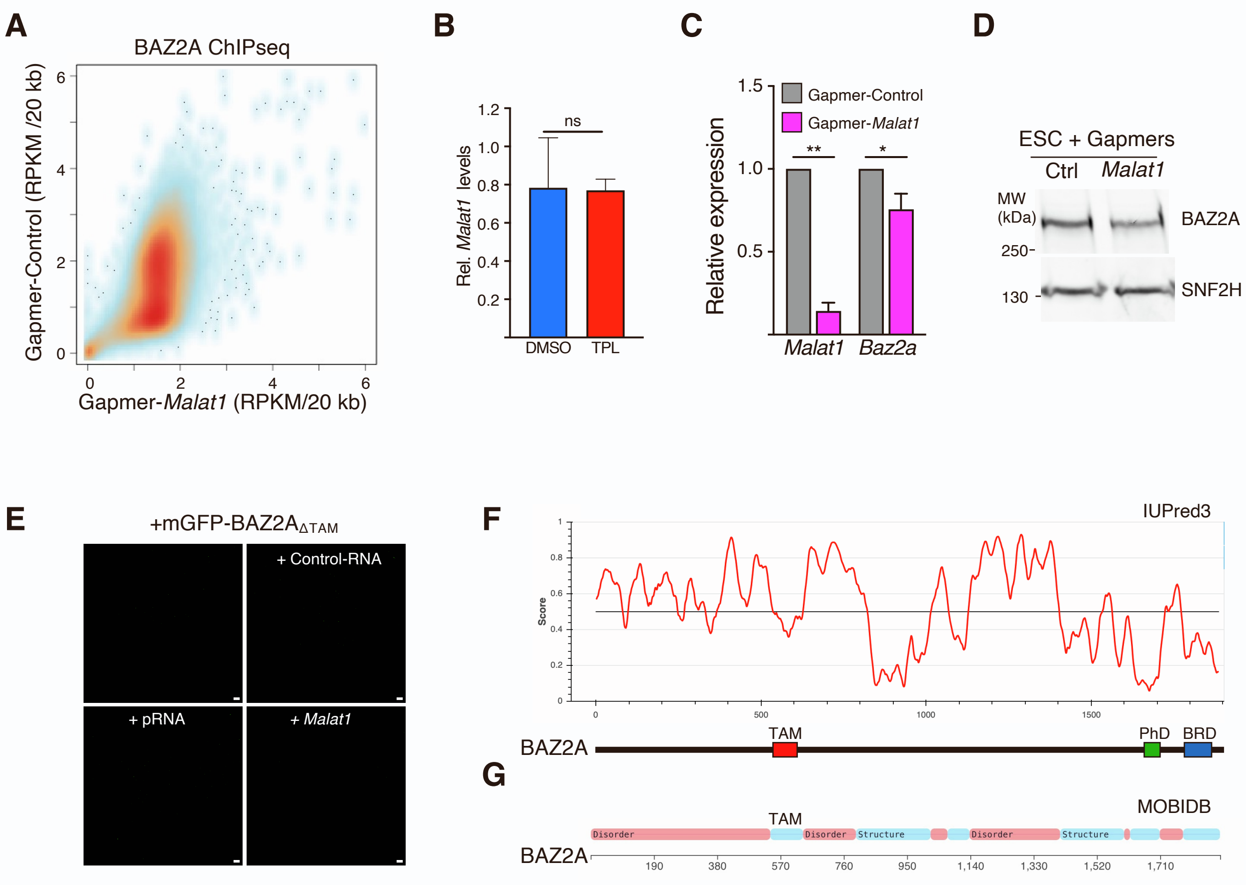

Figure S7 (Related to Figure 6 & 7)
BAZ2A associates with *MALAT1*

A. Scatter plot showing BAZ2A association with chromatin levels (reads/20 kb bin) between ESC+gapmer-Control and ESC+ gapmer-*Malat1*.

B. qRT-PCR showing *Malat1* levels in ESC+2i treated for 4 hours with triptolide. Data are from three independent experiments. Error bars represent s.d., and statistical significance (P-values) was calculated using the paired two-tailed t-test (ns: non-significant)

C. qRT-PCR showing *Malat1* and *Baz2a* RNA levels in ESCs treated with gapmer-control and gapmer-*Malat1*. Error bars represent s.d. and statistical significance (P-values) was calculated using the paired two-tailed t-test (* < 0.05; ** < 0.01).

D. Western blot showing BAZ2A protein levels in ESCs treated with gapmer-control and gapmer-*Malat1.* SNF2H was used as loading control.

E. Representative images of droplets of 500 nm BAZ2A recombinant mGFP-BAZ2A$_{N/\Delta TAM}$ and 2% PEG8000 in the absence or presence of 50 nM RNA-Control, pRNA, or *Malat1*. Scale bar represents 2 µm.

F, G. Prediction of IDR domains at BAZ2A using (**F**) IUPred3[73] and (**G**) MOBIDB[74].
